# Enhancing Wound Healing: A Comprehensive Review of Sericin and *Chelidonium majus* L. as Potential Dressings

**DOI:** 10.3390/ma17174199

**Published:** 2024-08-24

**Authors:** Ana Borges, María Luisa Martín Calvo, Josiana A. Vaz, Ricardo C. Calhelha

**Affiliations:** 1 Centro de Investigação da Montanha (CIMO), Instituto Politécnico de Bragança, Campus de Santa Apolónia, 5300-253 Bragança, Portugal; ana.borges@ipb.pt (A.B.); calhelha@ipb.pt (R.C.C.); 2 Grupo de Investigación en Desarrollo y Evaluación de Formas Farmacéuticas y Sistemas de Liberación Controlada, Facultad de Farmacia, Universidad de Salamanca, Campus Miguel de Unamuno s/n, 37007 Salamanca, Spain; 3 Grupo de Investigación en Fisiología y Farmacología, Facultad de Farmacia, Universidad de Salamanca, Campus Miguel de Unamuno s/n, 37007 Salamanca, Spain; marisam@usal.es; 4 Laboratório Associado para a Sustentabilidade e Tecnologia em Regiões de Montanha (SusTEC), Instituto Politécnico de Bragança, Campus de Santa Apolónia, 5300-253 Bragança, Portugal

**Keywords:** tissue repair, biomaterial, medical device, silk, greater celandine

## Abstract

Wound healing, a complex physiological process orchestrating intricate cellular and molecular events, seeks to restore tissue integrity. The burgeoning interest in leveraging the therapeutic potential of natural substances for advanced wound dressings is a recent phenomenon. Notably, Sericin, a silk-derived protein, and *Chelidonium majus* L. (*C. majus*), a botanical agent, have emerged as compelling candidates, providing a unique combination of natural elements that may revolutionize conventional wound care approaches. Sericin, renowned for its diverse properties, displays unique properties that accelerate the wound healing process. Simultaneously, *C. majus*, with its diverse pharmacological compounds, shows promise in reducing inflammation and promoting tissue regeneration. As the demand for innovative wound care solutions increases, understanding the therapeutic potential of natural products becomes imperative. This review synthesizes current knowledge on Sericin and *C. majus*, envisioning their future roles in advancing wound management strategies. The exploration of these natural substances as constituents of wound dressings provides a promising avenue for developing sustainable, effective, and biocompatible materials that could significantly impact the field of wound healing.

## 1. Introduction

The use of plant and natural extracts in wound dressings is a growing field of study motivated by the ability to promote wound healing [1,2,3]. Notably, Sericin, a protein derived from silk, and *Chelidonium majus* L., a botanical agent, have stood out as compelling candidates that offer a unique combination of natural elements that may revolutionize conventional approaches to wound care. This review article consolidates recent findings on the wound healing process and its treatments. As a result of major concerns and a higher incidence of conditions associated with wounds, particular consideration was given to the extracts Sericin and *C. majus*, namely, their properties, applications, and prospects. Some attention was also given to the possible creation of dressing containing these extracts.

Wound dressings have a long history, evolving since ancient times and undergoing continuous development [1]. The technology of wound dressings advanced significantly until the late 20th century, at which point a variety of commercial wound dressings, including hydrogels, foams, alginates, adhesives, patches, and meshes, became accessible. These varied shapes made it possible to transport both basic and complex medication molecules to the wound bed while also absorbing wound exudates, containing infection, and achieving the required vapor permeation rate [1,4,5,6].

Current research has focused on the role of herbal medicines and natural products in wound healing. In vitro and in vivo experiments show that natural extracts have significant antioxidant properties, promote wound healing, and have immunomodulatory and anti-inflammatory effects, accelerating the healing process [7]. Medicinal plants, synthetic and natural polymers, bioactive glasses (BG), and functional inorganic nanomaterials (NMs) are only a few of the materials that have been employed so far to treat wounds [8,9,10,11]. Researchers are exploring the potential of natural chemicals as improved wound dressings to improve this complex process [12,13,14,15].

As the body’s most delicate tissue, the skin is continuously vulnerable to threats from illnesses, injuries, and surgical incisions [8,16,17,18]. Described as a “silent epidemic”, wounds have a profound social and economic impact, negatively influencing the quality of life for millions globally [12,19,20,21]. Human life and health are at risk when the skin is significantly damaged; hence, regeneration must occur as soon as feasible to re-establish functions and return the body to balance [16,22,23].

Wound healing is a complex and dynamic process that relies on a myriad of biological factors and external interventions to restore tissue integrity [14,15,17,24,25]. It consists of multiple overlapping phases, including extracellular matrix (ECM) deposition, tissue remodeling, cell migration, and proliferation. To put it briefly, the creation of a fibrin clot ensures hemostasis and the early repair of the barrier integrity. The clotting cascade is triggered as soon as an injury occurs. In the meantime, inflammatory reactions are set off, boosting host defenses both locally and systemically against encroaching pathogens and for debris removal [24,26,27,28,29]. However, wound healing is typically not a fully regulated process. Abnormal wound repairs can result from a variety of circumstances at any time, including severe inflammation, burns, accidents involving extensive areas of skin tissue loss, infection, diabetes, and other conditions [27,28,30,31].

Scientific databases, such as Web of Science, Scopus, Scielo, Science Direct, and PubMed, were used to collect scientific articles and chapters for this review. The keywords “Wound healing”, “Sericin”, “*Chelidonium majus*”, and “Dressings” were used isolated and in combined forms.

## 2. Wound Healing

Wound healing is an intricate biological process that enables the body to repair damaged tissues and restore their normal structure and function. Whether the consequence of accidents, procedures, or long-term situations, a complex chain of cellular and molecular reactions that work together to seal the hole in tissue integrity is set off. Each phase of the intricate and dynamic wound healing serves a distinct function meticulously regulated by numerous signaling pathways and variables [3,13,32,33,34].

One health issue that human beings have struggled with for generations is wounds [35]. In addition to the various types (chronic wounds, acute wounds, surgical wounds, etc.) and treatments (dressings, films, natural products, etc.) already in existence, advances in modern medicine have facilitated the development of biomedical products, spreading their usage through mass production [19,35,36]. Significant improvements in wound care and infection control have been achieved as a result of these breakthroughs. Nevertheless, the persistence of delayed and non-healing wounds remains a significant concern for patients and medical professionals despite numerous studies on wound healing pathophysiology and the introduction of new techniques to promote healing [35].

Considering these aspects, it is crucial to enhance general awareness about wounds, the healing process, contributing causes, and available therapies [35], with a particular focus on natural products, which have recently gained popularity for a variety of reasons, including biocompatibility, anti-inflammatory and antibacterial characteristics, cost-effectiveness, and sustainability [31,35].

### 2.1. Wounds

As the largest organ in the body [3,7,19,37,38], the skin serves a variety of functions, including protecting against heat, light, injury, and infection, regulating body temperature, storing water and fat, serving as a sensory organ, and acting for social and cosmetic purposes [3,7,19,38]. The epidermis and dermis, which are connected by the foundation membrane, are the two primary structural layers that make up the skin. The subcutaneous tissue, the hypodermis, with the adipose tissue, is located beneath these layers [3,16,19,37,38]. Factors such as age, sex, anatomical location, and other factors can influence skin characteristics and properties, like the thickness and flexibility of the skin, for example [19].

A skin wound is a pathological condition initiated by chemical, mechanical, or thermal injury that causes the tissue to lose its ability to defend itself [3,19,36,38,39,40]. Swift and proper wound healing is crucial for restoring functional tissues and maintaining structural integrity after an injury [41] because it can pose a serious threat to the human body due to the potential for infection, fluid loss, and thermal dysregulation [40].

Wounds are a common occurrence throughout life [31] and can range from a little skin epithelial break to a severe injury to the subcutaneous tissue and nearby bone, muscle, and nerve systems [37,42]. It typically follows the pattern of bleeding, vessel contraction, blood clotting, cellular proliferation, formation of granulation tissue and new blood vessels, changes in the extracellular matrix (ECM), and a series of inflammatory reactions [37,42].

The adoption of a standardized categorization is challenging for the medical community [19]. According to numerous studies, wounds can be categorized according to several factors, including their source, depth, appearance, and healing characteristics [3,7,27,34,36,38,39,42,43].

Wounds can be classified into two primary groups, acute wounds and chronic wounds, depending on their healing characteristics [3,7,27,34,36,38,39,42,43]. Acute wounds are wounds that heal fully, with little to no scarring, and usually go through the normal, orderly stages of tissue restoration within a time frame of 8 to 12 weeks [3,11,27,38,42,43,44]. Most often, traumatic physical/chemical injuries or surgical treatments result in these kinds of wounds [36,38,44]. Chronic wounds, on the other hand, are those that exhibit delayed healing 12 weeks after the initial injury [3,11,27,38,42,43] and do not proceed through the typical stages of healing [11,27,39,42,43,44]. Repeated tissue assaults, underlying physiological abnormalities like diabetes, poor angiogenesis and innervation, or cellular migration are the main causes of such wounds [36,38,43,44]. Pressure ulcers, diabetic ulcers, venous ulcers, and arterial insufficiency ulcers are the four main groups of chronic wounds based on the aetiologies that cause them [27,38,43]. All of these non-healing wounds share certain characteristics in common, such as elevated levels of proteases, pro-inflammatory cytokines, persistent reactive oxygen species (ROS), senescent fibroblasts, prolonged infection, and dysfunctional or insufficient stem cells, even though their underlying causes may vary [38].

Wounds are also classified as either open or closed wounds depending on the underlying cause of their genesis [7,11,31,34]. When the skin is ripped, sliced, or punctured, there is an open wound [31]. Indicable bleeding occurs when blood flows from the body through an open wound [11]. Contrarily, a closed wound results from a contusion brought on by blunt force trauma, whereas burn wounds are brought on by fire, heat, radiation, chemicals, electricity, or sunlight. Closed wounds allow blood to leave the circulatory system but remain inside the body. Bumps and scrapes make it obvious [11].

Skin injuries can be further divided into three categories, taking into account the depth of the wound: superficial wounds where only the epidermis is affected, partial thickness wounds where both the epidermis and deep dermis are affected, and full-thickness wounds where the entire thickness of the skin, including subcutaneous fat, as well as deeper tissue, is destroyed [43]. Moreover, wounds can be divided into three categories according to the cause of the wound: surgical wounds (incisions, excisions, and surgically debrided wounds, incisions made during surgical procedures), pathological wounds resulting from comorbid medical conditions, such as pressure ulcers or diabetic foot ulcers, and traumatic wounds brought on by accidents, injuries, falls, cuts, or blunt force trauma [7]. Still, wounds can be categorized based on the mechanism of injury, such as laceration wounds, which are non-surgical injuries combined with other types of trauma that cause tissue loss and damage, puncture wounds that are caused by an object, like a nail or a needle, that punctures the skin, and abrasive wounds, which are caused by sliding onto a rough surface and causing abrasion. Avulsions are injuries when a piece of the skin and underlying tissue is torn away, and abrasions are injuries where the top layer of the skin, or the epidermis, is scraped off. This exposes nerve endings and causes a painful injury [11].

Wounds are medical conditions that lower patients’ quality of life, limit them socially, increase labor force loss and healthcare costs, and have an impact on both the patient and society [35,45]. The field of wound care and therapy spans a wide range of specialties. Treatment and care should be administered correctly for optimal wound healing [35]. There is still discussion about the need to create an ideal environment for wound healing and support healing holistically [35,37,46]. According to recent statistics, chronic non-healing wounds affect 2.5% of the entire world’s population and cause significant social and financial burdens [27,46]. Proper diagnosis of underlying causes is essential in managing wounds, taking into account both exogenous and intrinsic complicating variables [27,37]. The potential for preventing complications, as well as having a prognosis for morbidity, mortality, and quality of life, are two factors that contribute to the clinical value of accurate wound assessment and categorization. Additionally, it enables the development of a treatment strategy and enables the medical staff to determine whether the healing occurs within acceptable limits [19,27,37].

### 2.2. Process

**Figure 1:**
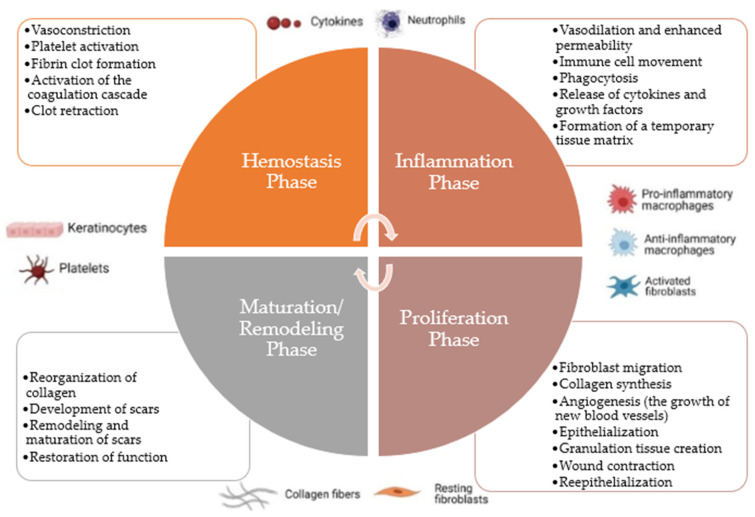
The different phases of the healing process. Scheme of the four dynamic phases of the wound healing process: (1) Hemostasis phase, which is regulated by platelets and fibrin; (2) Inflammatory phase, which is characterized by immune cells quickly removing debris; (3) Proliferative phase, which involves angiogenesis, fibroblast activity, and epithelialization; and (4) Remodeling phase, which involves matrix reorganization, wound contraction, and scar formation.

The development of a wound triggers a complicated, dynamic, multi-step process [32,35,47,48]. The process of wound healing comprises four continuous and overlapping phases (Figure 1): (i) hemostasis and coagulation; (ii) inflammation and swelling; (iii) proliferation and formation of tissues and blood vessels; and (iv) maturation and remodeling of tissue [37,39,42,47,48,49]. This process is crucial for restoring the function of the skin, involving the interaction of multiple factors, including cytokines, growth factors, blood, and extracellular matrix [34,37,39,48]. These phases take place in a controlled and accurate manner [32,35].

Despite the tissue type or the origin of the damage, the same events occur in the same order during the healing process [35]; however, the exact contribution of each of these stages to the wound healing process is varied and is primarily based on individual wound features [42]. Several factors may hinder the natural healing process and inhibit complete wound healing in this complicated process [45]. Compromised wound healing is an elaborate issue involving several dependent molecular and cellular mechanisms that are inextricably linked. A slight disruption in those processes results in the development of a chronic non-healing condition requiring a multifaceted approach to recovery [32,35,37,50,51].

#### 2.2.1. Hemostasis Phase

The hemostasis phase, the initial step in wound healing, begins as soon as the injury occurs. The primary objective of the hemostasis phase is to stop bleeding and prevent further blood loss from damaged blood vessels. The body’s internal environment must be maintained by this early response to avoid hypovolemic shock from severe blood loss [1,10,27,39,40,44,52,53]. This phase involves several complex processes to establish hemostasis (arrest of bleeding), comprising several complex processes, including vasoconstriction, platelet activation, fibrin clot formation, activation of the coagulation cascade, and clot retraction [1,7,10,16,27,38,39,40,44,52,53].

Injuries cause the blood arteries near the wound to constrict or narrow, which reduces blood flow and slows bleeding. Vasoconstriction is a quick and transient response caused by smooth muscle cells in the vessel walls contracting [7,39]. Blood’s minute cell fragments, known as platelets, are essential to hemostasis. When the blood artery is injured, platelets are exposed to collagen, a protein in the exposed connective tissue. Platelets become activated and adhere to the damaged site as a result of this exposure. After that, the active platelets emit chemical signals like thromboxane and serotonin that draw other platelets to the region and cause a platelet plug to form [38,52,53]. Activation of a complicated cascade of coagulation factors occurs as platelets group to form the platelet plug. In the end, this cascade transforms fibrinogen, a soluble blood protein, into fibrin, a fibrous protein. To stabilize the platelet plug, strengthen the clot, and create a more durable barrier that aids in stopping bleeding, fibrin creates a mesh-like network. A set of enzyme activities known as the coagulation cascade speeds up the clotting process. Both intrinsic and extrinsic routes combine to eventually activate thrombin, a crucial enzyme in the transformation of fibrinogen to fibrin [1,16,38,52]. Once the fibrin clot forms, platelets compress, bringing the wound’s borders together. This clot retraction aids in the healing process and helps to constrict the wound [52,53,54].

#### 2.2.2. Inflammation Phase

Following the hemostasis phase, the inflammatory phase constitutes the second step in wound healing [3,7,10,32]. It usually starts within hours of the injury and might linger for several days [7,10,32,40,42,55]. The inflammation phase’s major goal is to manage and confine any potential infection at the wound site, as well as to prepare the damaged tissue for the succeeding stages of healing [7,21,41,42,52]. It entails a complex and coordinated set of events mediated by immune cells, cytokines (cell-signaling molecules), and other inflammatory mediators [10,19,31,37,40,42,52,55]. This phase is marked by several events, including vasodilation and enhanced permeability, immune cell movement, phagocytosis, the release of cytokines and growth factors, and the formation of a temporary tissue matrix [5,19,26,31,32,40,44,52].

Blood vessels near the wound dilate in response to the damage, enhancing blood flow to the area. The increased blood flow at the wound site generates redness and warmth. Furthermore, blood arteries become more permeable, allowing immune cells and fluid-containing proteins to flow from the bloodstream into the tissue and cause swelling (edema) [3,41,52]. Immune cells such as neutrophils and macrophages are drawn to the wound site. Neutrophils are the first responders, and they are in charge of cleaning any germs or debris from the wound. Macrophages arrive later and play an important function in the removal of dead cells and the start of the tissue repair process. Phagocytosis is the process by which neutrophils and macrophages consume and destroy bacteria, injured cells, and other debris. This reduces the risk of infection and prepares the wound bed for the next stages of healing [5,16,19,26,32,37,52,55]. At the wound site, immune cells release cytokines and growth factors that govern numerous parts of the healing process. These signaling molecules attract more immune cells to the wound, boost fibroblast proliferation (cells that generate collagen), and promote angiogenesis (the development of new blood vessels) [32,52,53]. At the wound site, a temporary tissue matrix made of fibrin and other proteins is generated. This matrix functions as a scaffold for cell migration and as a guide for tissue repair [5,26,52].

While inflammation is an important and required part of the wound-healing process, excessive or persistent inflammation can stymie recovery and contribute to chronic wounds. As a result, the transition to the following phase of wound healing, the proliferation phase, is critical for successful tissue repair and regeneration [16,33,35,53]. Proper wound care during the inflammation phase involves managing infections, minimizing swelling, and maintaining a clean, moist environment for effective healing [3,7].

#### 2.2.3. Proliferation Phase

Succeeding the inflammatory phase, the proliferation phase represents the third step in the wound healing phase [3,32,53], which is typically initiated on the third day following the injury and lasts for a few weeks [10,32,37,41,52]. The primary goal of the proliferation phase is to repair and restore injured tissue by promoting the proliferation and migration of various cells essential for wound healing. It entails the coordinated interaction of several cell types and their functions [3,16,21,23,33,37,53]. In addition, crucial proliferative phase processes take place, including fibroblast migration, collagen synthesis, angiogenesis (the growth of new blood vessels), epithelialization, granulation tissue creation, wound contraction, and re-epithelialization [16,32,37,38,39,41,42,49,52].

The specialized cells known as fibroblasts travel to the site of the wound to produce the extracellular matrix (ECM). They release collagen, a crucial component of connective tissue, which creates the new tissue’s structural foundation. Collagen gives the tissue tensile strength and aids in wound healing [10,32,33,37,41,44,49,53]. New blood vessels start to enlarge around the wound during the proliferation stage, a process called angiogenesis. These fresh blood vessels stimulate cell growth and repair by supplying nutrients and oxygen to the growing tissue. At the borders of the incision, epithelial cells begin to move and multiply over the wound surface, progressively covering the wound region. Restoring the skin barrier and healing the wound depends on this procedure [7,10,32,37,41,44,49,53]. At the site of the wound, granulation tissue, a pinkish, granular tissue, forms. Fibroblasts, new blood vessels, and a transient collagen and protein matrix make up this structure [7,32,37,44,49,52,53]. The wound bed is filled with granulation tissue, creating an ideal environment for tissue repair. The edges of the wound contract as a result of forces applied by myofibroblasts, specialized contractile cells. This procedure pulls the wound edges closer together and helps to minimize the size of the wound [16,23,37,44,49,53]. Simultaneously, epithelial cells continue moving and covering the wound surface, granulation tissue develops, and wound contraction occurs. Sealing the wound and re-establishing the barrier-protecting function of the skin depend on this re-epithelialization process [20,32,37,41,42,44,49,53].

The proliferation stage of wound healing is an active and crucial phase. As new tissue grows and fills in the defect, the wound experiences substantial alterations throughout this stage. During this phase, it is essential to provide sufficient nourishment, oxygenation, and a moist wound environment to enable cell development and tissue repair [16,20,37]. To properly care for a wound during the proliferation phase, the developing tissue must be shielded from harm, the area must be kept moist, and the right dressings must be applied [33]. In this crucial stage of wound healing, timely interventions and appropriate wound management can promote successful tissue repair and reduce complications [3,39,42,53].

#### 2.2.4. Maturation/Remodeling Phase

Following the proliferation phase, the maturation phase, also known as the remodeling phase, represents the concluding stage of wound healing [31,33,37,40,41]. Depending on the size and severity of the wound, this phase can extend anywhere from months to years following the initial damage [10,32,41]. The newly created tissue goes through remodeling during this stage to increase its durability and functionality [10,31,33,42,53]. It involves several crucial processes, including the reorganization of collagen, the development of scars, their remodeling and maturation, and the restoration of function [10,37,41,52,53,55].

Strengthening the wound’s tensile strength involves the reorganization and realignment of collagen fibers initially laid down during the proliferation phase [32,52]. Matrix metalloproteinases (MMPs), a class of specialized enzymes, have an impact on collagen remodeling. Scar tissue gradually replaces the granulation tissue. Collagen fibers that make up a scar are more arranged and less cellular than the tissue during the early phases of wound healing. The scar tissue continues to change, becoming more streamlined and compact. The purpose of scar remodeling is to improve the tissue’s mechanical characteristics and bring it closer in consistency to the healthy tissue around it [16,32,52]. The scar lessens in color and elevation with time and may eventually merge with the surrounding skin. As the wound-healing process draws to a close, the scar ages and gets less visible. The wound’s functional characteristics get better as it matures as the tissue gets stronger and more flexible [32,52]. The repaired tissue could not fully achieve the same structural and functional qualities as the original tissue, though. It is vital to keep in mind that the maturation period is not always linear and might differ depending on the person and the type of wound. Age, general health, the extent of the incision, and the existence of underlying medical disorders can all affect how quickly and well tissue remodels [33,37,55].

Adequate wound care remains crucial during the maturation stage to ensure optimal healing results. It is imperative to take precautions to shield the developing tissue from harm, UV radiation, and too much tension. To promote scar maturation and reduce scar visibility, it may also be advised to use silicone-based dressings or gels on the scar [32,33]. It is important to keep in mind that scars are a normal part of the healing process and serve as a tribute to the body’s incredible capacity to repair itself after harm, even though the maturation period signifies the conclusion of the wound healing process. Scars may never completely disappear, although they often become smaller and less noticeable over time [32,33,41].

#### 2.2.5. Acute and Chronic Wound Healing

Following trauma or surgical injury, acute wound healing is the regular and predictable process that unfolds through the conventional stages of wound healing [1,10,27,29,40,48,52,56]. Conversely, treating and healing chronic wounds is more challenging due to persistent and delayed wound repair brought on by underlying medical disorders or outside influences [10,13,32,42,48,49,56]. To stop acute wounds from becoming chronic and to promote efficient wound healing in both types of wounds, proper treatment and early intervention are essential [13,32,52].

Due to the unique characteristics and underlying causes of acute and chronic wounds, the healing process varies greatly between them [1,13,29,43,48,49,52,56]. Acute wound healing proceeds naturally through the several stages that have already been highlighted [1,10,19,21,27,29,48,52]. The hemostasis phase, which follows platelet interaction with the extracellular matrix and broken collagen fibers, results in vasoconstriction and platelet activation. A temporary matrix enabling cell movement called a fibrin clot is created. Then comes the inflammatory phase, during which neutrophils and monocytes are drawn to the wound site from the bloodstream and infiltrate it to clear it of debris, pathogens, and injured tissue. In addition, growth factors and cytokines are released to promote cell development and start the repair process. The next step is proliferation, which includes re-epithelialization, angiogenesis, and the creation of granulation tissue, a second transient matrix abundant in fibroblasts and macrophages. At this time, the myofibroblasts start the process of wound edge contraction while the fibroblasts start to produce collagen. The maturation stage, which replaces the temporary matrix with the final one that is structured and rich in mature collagen, marks the end of the process [19,21,27,29,41,43,48,52,56].

However, the healing process for chronic wounds is marked by the following [1,10,21,40,43,48,52,55,57]:

- Persistent inflammation: With persistent inflammation and an excess of inflammatory cells, such as neutrophils and macrophages, chronic wounds frequently remain in the inflammatory phase. Chronic inflammation can prevent the body from mending properly [10,21,29,32,40,48,52];
- Delayed or impaired proliferation: Ischemia (poor blood supply), among other things, causes chronic wounds to show decreased cell proliferation and tissue healing. Reduced or delayed granulation tissue development prevents wound closure [10,21,40,48,52];
- Persistent infection: Chronic wounds are prone to lingering infections, which are frequently brought on by the growth of biofilms or bacteria that are resistant to antibiotics. These infections fuel persistent inflammation and foster an environment that is unfriendly to healing [10,21,32,40,48,51,52];
- Poor angiogenesis: The growth of new blood vessels, angiogenesis, is essential for supplying the wound site with nutrition and oxygen. It is frequently compromised in chronic wounds, which results in insufficient tissue oxygenation and further delays in healing [10,21,29,40,48,52];
- Excessive matrix metalloproteinases (MMPs): MMPs, enzymes that degrade collagen, may be present in greater amounts in chronic wounds. Excessive MMP activity can prevent the healing of wounds and the regeneration of new tissue [10,21,29,40,48,52];
- Repetitive Injury: due to variables like immobility or mechanical stress, certain chronic lesions, such as pressure ulcers or diabetic foot ulcers, are susceptible to recurrent harm. This ongoing trauma hinders recovery and makes the wound’s chronic nature worse [10,21,40,49,52,55,58].

Between acute and chronic wounds, the healing process differs primarily in that chronic wounds have ongoing inflammation, reduced cell proliferation, restricted angiogenesis, and the presence of elements like infection and recurrent injury that can cause delayed and halted repair [1,10,13,19,21,29,32,40,48,52,55]. Promoting good wound healing in both acute and chronic wounds requires addressing the underlying issues that prevent healing in addition to providing the necessary wound care [13,29,32,42,49].

### 2.3. Influential Factors in the Healing Process

The goal of wound management is to complete healing as quickly as possible while maintaining functional and aesthetic benefits [37]. For many years, wound treatment was predicated on covering the wound, with the materials used acting passively to promote healing. However, wound care has been revised in recent years as a result of a better understanding of the molecular and cellular processes involved in wound healing and wound prevention [19,37]. Wounds vary and can be described further by attributes such as blood flow, oxygen, infection, edema, inflammation, repetitive trauma and/or insult, innervation, wound metabolism, nutrition, previous injury handling, and systemic factors [19,37,56]. All of these characteristics can provide evidence of a wound’s genesis, etiology, and state [56]. Ultimately, given that the healing process is dynamic, it necessitates constant, systematic, and consistent evaluation involving a continuous reassessment of wound extent, type, and severity) [19,37,45,56].

So, various factors influence wound healing, which may be split into two categories: local factors (directly affecting the wound’s features) and systemic factors (individual’s general health or illness status, which influences his or her capacity to recover). Temperature, infection, and oxygenation are examples of local influences; however, age, diabetes, sex hormones, hereditary components, autoimmune illnesses, psychological stress, smoking, and obesity are examples of systemic factors [31]. An overview of the different factors is presented in Table 1.

Temperature is regarded as one of the most important elements for determining wound status since it can affect a variety of chemical and enzymatic actions, as well as the activities of cells and components involved in wound healing. Internal and external factors influence skin temperature, and changes in air temperature, surface wetness, body position, and blood flow can all have a significant impact. The temperature may influence wound healing by altering local blood supply, oxygenation, wound infection, inflammation, and lymphocyte extravasation [13,31,49].

Microorganisms that are normally found on the skin’s surface get access to the underlying tissues when the skin is injured. The condition of infection and replication status of the microorganisms is determined if the wound has inflammation (a normal aspect of the wound healing process and is required for the removal of pathogenic microorganisms), colonization (presence of replicating microorganisms on the wound without tissue harm), local invasion/critical colonization (intermediate stage that involves the growth of microorganisms and the initiation of local tissue responses), and/or spreading invasive infection (presence of reproducing organisms inside a wound that causes damage to the host). However, because microbial clearance is partial, inflammation might be sustained in the absence of successful cleaning. Pro-inflammatory cytokines such as interleukin-1 (IL-1) and TNF-alpha will contribute to both bacteria and endotoxins, extending the inflammatory process. If the wound is not treated properly, it might become chronic and refuse to heal. Furthermore, prolonged inflammation promotes an increase in matrix metalloproteases (MMPs), a protease family that can destroy the extracellular matrix (ECM). In conjunction with the increasing protease content, the level of naturally occurring protease inhibitors decreases. This shift in protease equilibrium may hasten the degradation of growth factors in chronic wounds [11,31,43].

Oxygen is required for practically all wound healing processes and is required for cell metabolism, specifically the creation of energy via ATP. It protects wounds from infection, induces angiogenesis, promotes keratinocyte differentiation, migration, and re-epithelialization, boosts fibroblast proliferation and collagen synthesis, and aids wound contraction. Because of absorption by metabolically active cells, the early wound microenvironment is depleted of oxygen and extremely hypoxic. Several systemic illnesses, including aging and diabetes, will result in decreased vascular flow, laying the scenario for inadequate tissue oxygenation. In the sense of recovery, this combination of insufficient perfusion results in a hypoxic wound [11,31].

The age of the patient influences wound healing; the danger of excessive wound healing is greater as one ages. During fetal wound healing, neurovascular and dermal architecture recover. Scarring is more likely in fetal skin than in adult skin. Migration of neutrophils and macrophages occurs early, and there are fewer inflammatory cells. Aging causes a temporary delay in wound healing in stable older persons but is not a serious handicap in terms of healing consistency [11,31].

Wound healing is greatly influenced by genetic factors. Men have different inflammatory responses and take longer to heal acute dermal wounds than women. Delays in healing are caused by inborn genetic illnesses such as Down syndrome and Ataxia-telangiectasia. Immune system illnesses such as hemoglobin synthesis disorders, vasculopathy, connective tissue diseases, and progeroid syndromes can be caused by gene mutations, chromosomal aberrations, and other genetic abnormalities. Klinefelter patients (chromosomal abnormalities) are more likely to have varicosis and thrombosis, with 13% suffering venous ulcers. Sickle cell anemia is caused by a malfunction in hemoglobin production [31].

Patients with illnesses have significantly larger wounds that take significantly longer to heal than the general populace. Leg ulcers have been reported in several autoimmune illnesses, particularly those related to connective tissue abnormalities. Ulceration is most common in rheumatoid arthritis and systemic lupus erythematosus. Furthermore, foot ulcers are common in people with rheumatoid arthritis, and the much longer time it takes for them to heal raises the risk of infection [31]. Coronary heart disease, peripheral vascular disease, stroke, and diabetes mellitus are only a few of the chronic disorders that might delay wound healing [11]. Diabetes-related wound healing delays involve a wide range of molecular activities, including sustained pro-inflammatory cytokine activity, impaired angiogenic response, microvascular complications, impaired macrophage and neutrophil development, keratinocyte and fibroblast migration, and proliferation [13,31]. Patients with illnesses should be constantly monitored throughout their course of care to have the best strategy [11].

Psychological trauma has a strong modulating effect on the individual suffering from a wound’s attitude, behavior, and general health state cytokines [11,31]. Stress reduces the levels of the pro-inflammatory cytokines IL-1, IL-6, and TNF- near the wound. It also reduces cytokine IL-1 release, interfering with the well-regulated inflammatory process of physiologic wound healing. Stress may impair wound healing by increasing glucocorticoids, which reduce immune cell proliferation and decrease wound site synthesis of IL-1, IL-1, and TNF cytokines [31,43].

Nutrition has an impact on wound healing. The most obvious effect is that hunger or specific nutritional deficiencies following trauma and surgery can have a significant impact on wound healing. Patients with chronic or non-healing wounds, as well as those with nutritional deficiencies, require additional nutrients. Energy, carbs, proteins, lipids, vitamins, and minerals all have an impact on the healing process [11]. Obese people are predisposed to several significant illnesses, including delayed wound healing, which may be caused by hypoperfusion and ischemia symptoms in subcutaneous adipose tissue. As a result, if the tissue around the wound is deficient in oxygen, the oxygen-dependent cellular repair mechanisms are deficient. Hypovascularity, which is frequent in obese patients, causes insufficient perfusion and increases the risk of infection by limiting immune cell penetration into the wound field [11,31].

Moreover, other important factors that affect the healing process were mentioned, such as vascular insufficiency (various wounds or ulcers—such as arterial, diabetic, pressure, and venous ulcers—can affect the lower extremities) [11], cellular senescence (terminal response of cells that exits the cell cycle under stress and damage, affecting tissue homeostasis and affecting tissue repair and regeneration) [43], smoking (disrupting microcirculation, reducing inflammation, and affecting fibroblast migration, proliferation, and collagen synthesis) [31], angiogenesis (chronic wounds have higher anti-angiogenic molecules and lower angiogenic factors, while excessive proteolysis of angiogenetic factors contributes to cell apoptosis and insufficient nutrition for repair) [43], pH (crucial indicator of wound status, affecting physiological processes like collagen formation, inflammatory response, and angiogenesis) [13,49,59], and uric acid (UA) (biomarker associated with bacteria colonization and is primarily found in wound fluids and exudates—a sharp decrease in UA concentration at the injury site can indicate bacterial infection) [13].

Improving the wound microenvironment is crucial for healing [60]. When considering the numerous treatments available, it is crucial to keep in mind the previously mentioned aspects that have an impact on the healing process [44,49,60]. By encouraging tissue regeneration and reducing infection, scarring, and discomfort, the moist wound environment has been demonstrated to specifically offer the optimal circumstances for quicker and better wound healing [36,60].

### 2.4. Treatment

Persistent wounds reduce the quality of life, and the cost of care is seen in morbidity and even mortality, as well as from a psychological standpoint and in the lengthened hospital stay. The medical system would be relieved of a significant financial load as well as dramatically reduce morbidity and death if wound healing were successfully managed. Due to all of these factors, the area of wound care will greatly benefit from the development of new technologies used in conjunction with established techniques to speed up the healing process [36,49,56,61].

**Figure 2:**
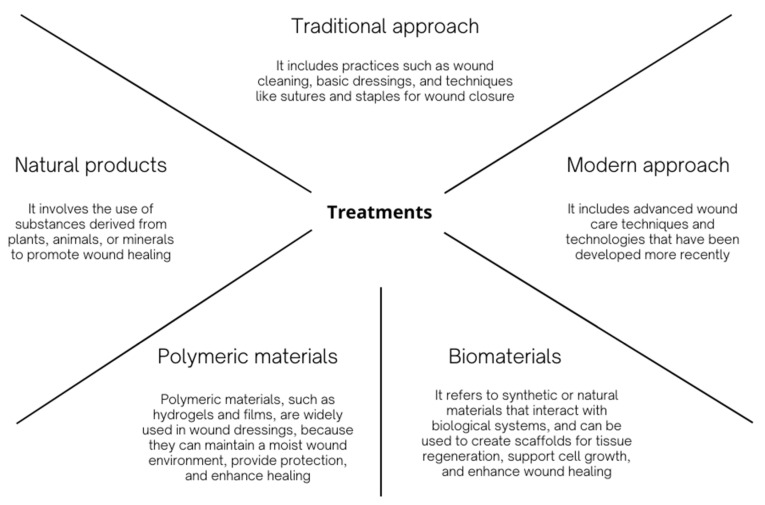
Treatments. Figure delineating diverse medical treatment strategies for wounds: Traditional approach—emphasizing hygiene and dressings, Modern approach—incorporating advanced wound care techniques, Biomaterials—synthetic or natural materials, Polymeric materials—including hydrogels, films, and Natural products—use of plants, animals or minerals.

Further, each phase’s therapy is an efficient strategy for promoting wound healing, but there are still a lot of challenges because of the variety of wound types and the many wound healing techniques [47,56,61]. Therefore, choosing an appropriate medical treatment strategy for a particular wound is desirable for wound healing [47]. To speed up the healing process, traditional or modern methods are required (Figure 2) [41].

#### 2.4.1. Traditional Approach

Traditional wound healing techniques cover a wide spectrum of time-tested procedures and treatments that have been used by various tribes and cultures throughout history. These techniques, which are frequently based on age-old knowledge and handed down through the generations, have helped lay the groundwork for wound care and are still applicable in some situations [10,41]. Traditional wound healing techniques continue to be important, especially in environments with limited resources or a rich cultural heritage. This is true even when modern medical breakthroughs have introduced creative alternatives [10,52,62].

Various plants and herbs have been used throughout history as therapeutic agents by numerous cultures. Aloe vera, turmeric, and honey are a few examples of substances that have been employed for their antibacterial and anti-inflammatory properties. To speed up healing, herbal poultices are applied immediately to wounds after being produced by crushing or masticating plant components [10,52]. Additionally, applying warm compresses or ice packs might help lessen discomfort and swelling brought on by wounds [55]. Different tribes have created distinctive bandaging techniques using available resources like leaves, bark, or linen. These bandages could be both protective and symbolic [10,52,62]. Additionally, some cultures use light massage and manipulation of the area close to the incision to enhance blood flow, lessen swelling, and accelerate healing [55]. Also, traditional dietary practices high in vitamins, minerals, and protein boost general health and promote wound healing [55].

#### 2.4.2. Modern Approach

Modern approaches to wound healing include a variety of cutting-edge medical procedures, devices, and treatments that have been developed through technological advancement and scholarly study [36,41,50,56]. These techniques are intended to hasten and optimize wound healing, enhance results, and reduce problems. The field of wound treatment has substantially improved thanks to contemporary approaches to wound care that are based on empirically supported practices [36,41,56].

There are numerous examples in this subject, including sophisticated dressings, scaffolds, matrices, novel medicines, and technology [10,36,41,56]. Bioactive dressings include substances like growth factors, collagen, or silver nanoparticles that hasten tissue regeneration and foster an environment that is favorable to healing [36,46,50,56]. Other examples include hydrogels and hydrocolloids, which keep a wound moist, enable autolytic debridement, and act as an infection barrier [36,46,50,56,63]. Foam and alginate dressings are used for wounds with significant exudate because they are very absorbent and offer support and protection [36,50]. Additionally, Negative Pressure Wound Therapy (NPWT) uses specialized dressing and vacuum equipment to impart negative pressure to the wound. It improves blood flow, drains extra fluid, encourages wound contraction, and encourages the growth of granulation tissue [36,52,57]. In addition, skin substitutes made of living cells and extracellular matrix components are utilized to cover wounds with poor healing capacity and encourage tissue regeneration [36,41,56]. Topically, growth factors and cytokines can be applied to promote wound healing, angiogenesis, and cell proliferation [36,41,56,61]. Patients who receive Hyperbaric Oxygen Therapy (HBOT) breathe pure oxygen in a pressurized chamber, which improves the oxygenation of tissues and speeds up the healing of wounds, especially those that are chronic or stubborn to heal [41,56]. Additionally, Negative-Pressure Wound Closure (NPWC) in conjunction with surgical methods can lessen tension on wound margins and enhance closure results [41,55]. The surgical transplantation of healthy skin or tissue, known as skin grafts and flaps, can cover wounds, promote healing, and enhance cosmetic results [36,41,52,56,61]. Bioengineered Scaffolds and Matrix Materials are utilized as scaffolds to enhance cell adhesion, encourage tissue regeneration, and direct tissue growth. To increase angiogenesis, stimulate cell activity, and speed up wound healing, electrical currents can be applied to wounds [36,41,52,56,61]. Applications of concentrated platelets or growth factors are used in biological agents, Platelet-Rich Plasma (PRP), and growth factor therapy to promote tissue repair. Advanced technologies, including digital imaging, ultrasound, and spectroscopy, aid in evaluating the condition of wounds, tracking their healing, and directing treatment choices [36,41,55,56,64]. Digital platforms allow healthcare providers to monitor wounds remotely, allowing for quicker interventions and a reduction in the number of in-person visits [36,41,55,56].

With continued study and technological developments, modern wound healing techniques are always changing. These methods seek to offer specialized and efficient treatments for distinct wound types, providing improved patient outcomes and a higher standard of living [36,41,46,48,50,52,56].

#### 2.4.3. Natural Products

Natural products for wound healing are compounds that come from plants, animals, minerals, or other natural sources that have qualities that help in tissue regeneration and wound healing [10,31,41,44]. These items continue to be prized for their possible therapeutic properties despite having been utilized for millennia in conventional medical procedures. Utilizing nature’s inherent healing abilities, natural products provide an alternative to or a supplementary method to contemporary wound treatment [10,31,40,44,65].

Honey has been utilized for its antibacterial, anti-inflammatory, and wound-healing properties since ancient times. Due to its high sugar content, a hypertonic environment is produced, which prevents bacterial growth and lowers the risk of infection. Additionally, honey encourages the production of cytokines and growth factors, aiding in tissue regeneration and wound healing [65]. Aloe vera gel, which is made from the plant’s leaves, is prized for its calming and anti-inflammatory qualities. It promotes a favorable environment for healing by easing discomfort, redness, and swelling and moisturizing the wound bed [41,44,65]. Turmeric contains a substance called curcumin, which has anti-inflammatory and antioxidant properties. By regulating the immune response, turmeric treatment can hasten wound healing and lower the risk of infection [31,41,65]. Fatty acids, included in coconut oil, moisturize and encourage a moist wound environment. Its antibacterial qualities can aid in healing and infection prevention. Strong antibacterial and antiseptic effects can be found in tea tree oil. To clean cuts and stop bacterial colonization, it can be applied topically [65]. Antibacterial and anti-inflammatory properties are present in lavender oil. It might ease skin irritation, lessen pain, and accelerate recovery [31,65]. Due to its anti-inflammatory and wound-repairing characteristics, calendula, which is produced from marigold flowers, has been used for wound healing. It can lessen the formation of scar tissue and aid in the promotion of granulation tissue [31]. Bees that pollinate the manuka bush create manuka honey, which has special antimicrobial ingredients. Its use can aid in battling bacteria that are resistant to antibiotics and speed up the healing of wounds. Natural antibacterial, anti-inflammatory, and antioxidant qualities can be found in the resinous material called propolis, which bees gather. It can help with tissue regeneration and wound sanitation [31]. Other examples include several plants, such as *Achillea millefolium* (yarrow) and *Centella asiatica* (gotu kola), which contain substances that promote collagen synthesis and angiogenesis while promoting wound healing [31,40,44].

Even though using natural products has some promising benefits, it is still best to use caution and talk to a professional before doing so, especially if you have allergies or other sensitivities. By incorporating natural items into wound care, the overall approach to managing wounds can be improved and given more options for healing [44,65,66].

#### 2.4.4. Biomaterials

Modern wound healing relies heavily on the use of biomaterials, which provide creative ways to speed up the healing process and enhance wound closure and tissue regeneration. These substances are intended to interact with biological systems, increasing angiogenesis, cellular activity, and general wound healing. Biomaterials offer a flexible framework for cutting-edge wound treatment, which contributes to better results and increased patient well-being [5,37].

Biomaterials act as scaffolds for tissue growth and cell adhesion, encouraging cell migration and division as well as the growth of new tissues. Additionally, they promote angiogenesis, which nourishes and oxygenates the wound area. Biomaterials can enhance cellular responses and lower the risk of infection by releasing growth factors, cytokines, or antimicrobial drugs into the wound bed. Furthermore, they promote cellular migration, shield the site from desiccation, and maintain a moist wound environment. To guard against bacterial colonization and illness, several biomaterials contain antimicrobial coatings or built-in defenses. Large wounds can be covered with biomaterials like dermal matrices and skin substitutes, which can facilitate tissue regeneration, remodeling, and collagen alignment. They offer customization possibilities, such as simulating the features of genuine tissue or dispensing particular drugs, which can be tailored to match the individual needs of patients and wounds [32,43,61].

Collagen-based scaffolds, synthetic polymers, hydrogels, and tissue-engineered skin substitutes are a few examples of biomaterials utilized in wound healing. These materials are the result of an in-depth study, and they have characteristics that are compatible with patient needs and wound healing principles. Although there are many potential advantages of using biomaterials, the best wound healing results require careful consideration of parameters like biocompatibility, degradation rates, and mechanical qualities [5,35].

The use of biomaterials in wound care exemplifies the growing convergence of engineering and medical research, giving medical personnel strong tools to handle challenging wound issues and ultimately enhance the quality of life for patients [5,37].

#### 2.4.5. Polymeric Materials

By providing adaptable solutions that improve wound management, increase healing conditions, and provide focused therapeutic interventions, polymeric materials have transformed the way wounds heal. These materials, which are frequently made from artificial or natural sources, can be specially designed to target particular wound features and encourage tissue regeneration. Polymeric-based wound care solutions have become an essential part of contemporary wound healing procedures and offer a variety of advantages for both acute and chronic wounds [36,37,44,67].

By encouraging cellular migration, angiogenesis, and the creation of granulation tissue, polymeric wound dressings, such as hydrogels and hydrocolloids, produce a moist wound environment that encourages healing [37,44,67]. Controlling moisture promotes autolytic debridement and prevents wound desiccation. Hydrocolloid dressings and other absorbent polymers are excellent at managing excessive wound exudate while keeping the surrounding area wet [36,37,44]. Polymeric materials can be designed to encapsulate and release therapeutic substances directly at the site of a wound, such as antibacterial medicines or growth hormones. By delivering focused and sustained treatment, controlled medication delivery improves wound healing [37,44]. To provide a favorable environment for cell adhesion, proliferation, and tissue regeneration, polymers can be created to simulate the extracellular matrix. They support the development of granulation tissue and promote angiogenesis [37,44], as well as both adhesive and non-adhesive wound dressings that stick to or protect the wound bed. Some polymers have inherent antibacterial characteristics that can aid in preventing bacterial colonization and illness [37,44]. Based on the features of the wound and the requirements for healing, polymers can be created to exhibit particular qualities, such as elasticity, flexibility, or rigidity. To protect the wound from external contaminants and to allow for gas exchange, polymeric films or barriers are used [36,37,44]. Soft and cushioning polymers, such as foam dressings, lessen discomfort and damage to the wound site while aiding healing [37,44]. Polymers are utilized to make bandages and dressings that adapt to the shape of a wound or a hard-to-reach location. Polymeric materials are used to make absorbable and non-absorbable sutures that help with tissue approximation and wound closure [36,37,44].

The biocompatibility, safety, and efficacy of polymeric materials used in wound healing are ensured through intensive research and development. These resources provide medical personnel with adaptable tools to handle a variety of wound forms and patient needs. The potential for creative wound care solutions is growing as polymer science develops, which will improve patient quality of life and the effectiveness of wound healing [36,67].

## 3. Dressing Creation

Medical devices are materials, tools, apparatus, appliances, or software that are used to track the health of humans, according to the World Health Organization [36]. Based on these qualities, as well as the many kinds of wounds, wound dressings are categorized as medical devices. Although the standards for classifying wound dressings are comparable in most countries, there are still variations in the processes for submitting dossier content and evaluating it. Due to common understanding and shared regulatory elements, the harmonization law expedites the licensing and marketing of medical devices in other nations. Technology and human understanding advances have made it easier to manufacture medical devices, which has resulted in the development of many generic devices for a wide range of illnesses. The global health organization continues to face significant obstacles in developing universal dressing recommendations due to the size and kind of wounds [4,36,68].

Wound healing is one of the most intricate pathological healing processes in the human body, and it is influenced by several factors [34,36,57]. A proper dressing acts as a supporting structure, preventing infection, supplying essential nutrients, and speeding up the healing process [34,36,69].

The financial burden of wound care is challenging due to its widespread use across various professions and settings [4,39,70,71]. Addressing the length of recovery times, dressing changes, and frequency of problems like infection can improve wound care management. Shorter recovery times reduce the use of materials, dressing changes, and healthcare provider hours. Reducing problems reduces surgical procedures, hospital stays, and resource consumption. Creating and using dressings that address these areas can reduce wound care costs and the number of wounds requiring treatment [39,68,69].

The growing number of wounds emphasizes how crucial it is to create cutting-edge solutions that aid in healing [4,25]. Wound care has changed significantly over time. Gone is the belief that a dry wound environment would promote healing; instead, moisture retention is now given priority when creating fresh wound dressings. The kind of wound dressings that were utilized also changed in tandem with this movement in knowledge [25,27,57,69].

More advanced materials, such as hydrogels, hydrocolloids, foams, films, etc., have replaced the more conventional wound dressings, such as gauze, lint, and cotton wool, which were only intended to cover the wound. These materials aim to strengthen cellular migration, encourage angiogenesis and the synthesis of new tissue, permit gas exchange from and to the wound, maintain proper tissue temperature, guard against bacterial infection, be easily removed after healing or in between dressing changes, promote autolytic debridement, and be sterile, non-toxic, and hypoallergenic [25,27,70].

The addition of antimicrobial agents, including antibiotics, natural products, and antiseptics, to wound dressing formulations is one of the more recent benefits, and it is an indirect way to reduce inflammation by clearing bioburden. Additional data for a more thorough examination of the various kinds of antimicrobial agents and dressings can be found in many reviews [25,57].

Today’s market offers thousands of various wound dressings, some with differing compositions but all claiming the same benefits. Physicians face an increasingly complex problem when selecting an appropriate dressing due to the multitude of alternatives available [25,72]. The type and location of the wound, the amount of exudate, the integrity of the surrounding skin, and whether the wound is infected or has stalled in the inflammatory phase of healing are just a few of the variables that must be taken into consideration when selecting an appropriate dressing to promote faster healing [25,36,73,74].

An optimal wound dressing should include the following qualities: preservation of a wet wound region, water resistance, the permeability of gas, avoidance of bacterial infection and disease transmission, prevention of desiccation and excessive exudate absorption, easy to change or remove biocompatible, biodegradable, and non-toxic, and low cost [3,6,27,39,57,75,76,77,78].

There are two main categories of wound dressings: traditional and modern [3,36,64,79]. Traditional wound dressings have been around since the 19th century and have been widely utilized because of their low cost, ease of application, and straightforward production process. Lint, plasters, gauze, and bandages are examples of this type of dressing. While they shield the site from outside pathogens, they are unable to regulate the amount of moisture they absorb from the wound, making it too dry for prompt healing. Furthermore, if there is an excessive amount of wound drainage, they could stick to the site and require difficult and painful dressing removal [3,27,33,39,64,79,80,81,82]. On the other side, modern wound dressings have advanced significantly in recent years and are now available in a variety of kinds, each intended to treat specific wound types and healing requirements. Because of their great absorption, hydrocolloid dressings are perfect for wounds with moderate to heavy exudate. Conversely, foam dressings are designed for wounds that produce a lot of exudates and are absorbent. Seaweed-based alginate dressings are extremely absorbent and are usually applied to wounds that discharge a lot of fluid. Hydrogel dressings are advised for wounds with mild to moderate exudate because they help to maintain a moist wound environment. Transparent film dressings are commonly used for superficial wounds or as a secondary dressing to hold other dressing types in place. They function as a barrier against moisture and bacteria [3,27,33,39,64,79,81,83].

Nevertheless, there is still a lot of interest in enhancing these materials’ functionality and effectiveness [84,85,86]. In response, novel dressing materials made of natural ingredients—like antiseptic plants—that not only shield the wound from infection but also promote skin regeneration have been or are in the process of being created [22,80,84,86]. The successful study and development of an eco-friendly wound dressing made using green methods may help minimize a significant amount of waste produced by the production of traditional dressings, given the increased requirements for wound dressings [85].

Numerous natural biomaterials are available for use as wound dressings. Polymers and nanobiomaterials are two categories of natural biomaterials. Once more, the polymers are divided into synthetic and natural (biopolymers) categories [87]. While synthetic polymers are made from nonrenewable petroleum sources, natural polymers are synthesized from renewable sources, such as plants and animals [22,81,87]. When it comes to producing bio-composite material, biopolymers—which might be polysaccharides or proteins—offer several benefits over synthetic polymers, including superior biocompatibility, biodegradability, and quick manufacturing [22,86,87,88].

The use of polymer-based membranes is one of the most well-known methods for wound healing. To date, several natural biomaterials, including collagen, alginates, chitosan, and gelatin, have been presented to facilitate the healing of wounds. A growing number of investigators are becoming interested in other natural biomaterials, like the silk proteins (silk fibroin (SF) and silk sericin (SS)), because of their exceptional mechanical qualities, excellent biocompatibility, permeability to water and oxygen, controllable proteolytic degradation, and low immunogenicity [89,90,91,92].

Natural polymers, like sericin, are used in wound dressings for skin regeneration and wound healing. Sericin’s properties make it suitable for various biotechnological and biomedical purposes [9,78,93,94,95,96,97,98,99,100]. Sericin is a potent antioxidant that aids in wound healing by promoting keratinocyte and fibroblast proliferation and collagen production. It can also be used as a wound dressing or cream to control infection, treat burns, reduce scarring, and prevent infection. Sericin-containing substances can prevent prolonged inflammation in chronic wounds and accelerate healing by turning the healing process into a proliferative phase. Their porous, 3D structure is ideal for wound-healing dressings and is cost-effective [78,93,94,95,99,101,102,103,104]. Combining them with naturally occurring bioactive substances, like plants, can improve their capacity to heal wounds [46,78,105].

In response, novel dressing materials made of natural ingredients like plants that not only shield the wound from infection but also promote skin regeneration have been or are in the process of being created [84]. One of the many examples is *Chelidonium majus* L. Greater celandine is a medicinal herb whose potential uses in wound treatment and dressing manufacture have been investigated. It contains bioactive components, including alkaloids and flavonoids, which have anti-inflammatory and antibacterial activities, albeit their use in wound care is not as widespread as that of certain other botanicals. These characteristics imply that *C. majus* extracts might be taken into consideration for inclusion in wound dressings to promote the healing process. Interest in the plant’s potential utility in making dressings that support a favorable wound environment is sparked by its historical usage in herbal therapy, especially for skin disorders [84,106,107,108,109].

**Figure 3:**
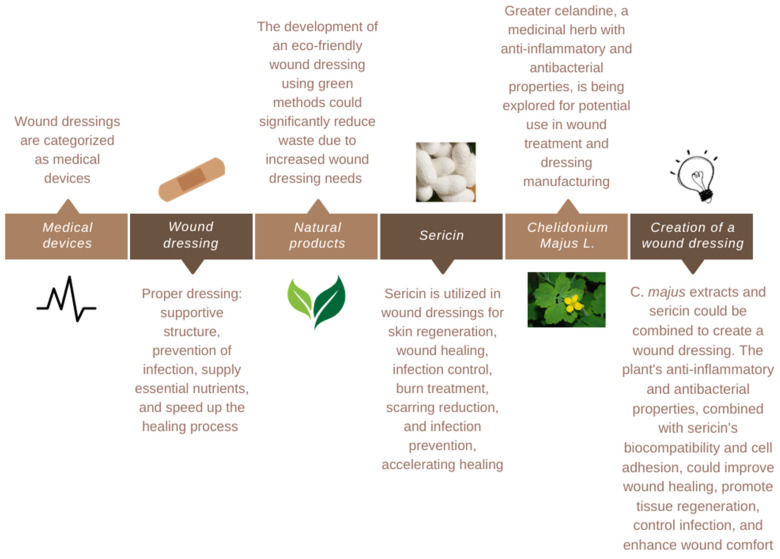
Schematic representation of an innovative wound dressing formulation. Conceptual illustration of a novel wound dressing formulation merging the healing virtues of *C. majus* and the regenerative potential of Sericin.

So, a fascinating synergy of natural components with possible therapeutic effects is presented when extracts from *Chelidonium majus* L. are combined with sericin to create a wound dressing [78,110]. The suggested dressing for wounds will offer a novel combination of sericin and extracts from *C. majus*, a first in the field of wound care biomaterials. Alkaloids and flavonoids, which are recognized for their anti-inflammatory and antibacterial qualities, are among the bioactive components contributed by the plant, which has a long history in traditional medicine. *Chelidonium majus* L. extracts are included to take advantage of these inherent qualities and create a setting that promotes faster wound healing. This botanical base will be enhanced by the silk-derived protein sericin, which is biocompatible and has been shown to encourage cell adhesion and proliferation. By promoting tissue healing and cellular regeneration, the resultant composite dressing will not only address current wound-related issues but also anticipate a comprehensive treatment strategy. This innovative dressing idea presents a paradigm change in wound care by fusing the precision of sophisticated biomaterials with the healing knowledge of nature. It arises at the nexus of botanical science and biomaterial innovation—where innovation, science, and nature come together to provide the best possible therapeutic result (Figure 3) [36,110].

While there may be therapeutic advantages to using sericin and *C. majus* together in a wound dressing, there may also be drawbacks to take into account. Making sure that sericin and *C. majus* extracts are stable and compatible in a cohesive dressing formulation is a difficult task. The overall effectiveness and safety of the dressing could be impacted by the chemical reactions between these ingredients. Furthermore, some people may be at risk due to the allergenic potential of sericin and the possibility of skin sensitivity to chemicals in *Chelidonium majus* L. Furthermore, the efficacy of the dressing may not always be uniform due to variations in the amounts of active chemicals resulting from natural plant extract variability [36,71,78,80,108]. A contrast of the effectiveness and safety of this innovative dressing with other common drug delivery systems used in wound care is presented in Table 2.

The most often used medical equipment that is essential to healthcare systems is wound dressing [36,39]. Establishing global regulatory guidelines for recently produced dressings will require more research [36,39]. As a result, choosing the right dressing needs to take into account how the wound is healing or deteriorating. As a result, it is necessary to continuously update and apply current regulatory principles throughout the production process [36,39,68].

Furthermore, research on developing wound dressings using natural compounds like polymers and plants is crucial for enhancing wound healing and optimal properties [86]. Further studies are needed to evaluate safety and effectiveness, considering wound bed characteristics like pH, temperature, and moisture [83,86]. Advanced techniques like electrospinning and 3D printing can be used to create controlled dressings [27,86]. Clinical trials are needed to evaluate the potential of these dressings in treating infections and addressing issues like pain, moisture loss, and cost [83,86].

## 4. Sericin

The secretion process of various arthropod lineages, including wasps (nests), silkworms (cocoons), and spiders (webs), results in the production of silk, a natural biopolymer. Sericin (15–35%) and fibroin (65–85%) make up its two main parts [50,77,113,114,115,116]. The majority of the structure is made of fibroin, and the glands of silkworms (such as Bombyx mori, Bombyx tangerines, and other species) create sericin, a naturally occurring gum-like, water-soluble, and adhesive protein (20–400 kDa) [50,77,113,115,116,117].

Sericin, a hydrophilic protein that holds the silk strands together to maintain the cocoon’s stickiness, is thought to be dumped in the effluent in the amount of 50,000 tonnes out of the 400,000 tonnes of dried cocoons produced globally, posing an environmental risk [94,114,115,118,119,120,121]. Despite its biological properties such as biocompatibility, immunocompatibility, biodegradability, anti-inflammatory, antibacterial, antioxidant, and photoprotective, among others, sericin is removed during the degumming of silk. During this process, different chemical, enzymatic, or hydrothermal methods are used to prepare smooth, soft, and lustrous silk filaments from silkworms’ cocoons for the textile industry, which are primarily discarded in wastewater. Sericin removal presents some challenges, including the high volume of oxygen required for its degradation by microorganisms and the burden of economic issues, especially in countries where silk farming is extensively practiced, such as India and China. These challenges are in addition to the environmental concerns posed by different pollution discarded in wastewater [54,80,94,112,115,118,119,122,123,124,125,126,127,128,129].

Indeed, there are 18 amino acids in sericin, the three most significant being serine (32%), aspartic acid (18%), and glycine (16%). This protein comprises 12.2% non-polar amino acids, 42.3% polar amino acids, and 45.8% hydroxy, amino acids (serine and threonine) [54,96,111,113,114,115,116,117,120,124,130,131]. The biological properties of these polar chemical groups are a result of their organic makeup, solubility, and structural organization. However, they also enable the creation of blends with other polymers through crosslinking, copolymerization, or blending, increasing the mechanical resistance of sericin-based biomaterials [54,77,80,115,116,118,120,127,128,132,133,134].

The US Food and Drug Administration (FDA) has approved the use of sericin for medical purposes [75,90,115,116,118,135,136]. It can be incorporated into hydrogels, films, sponges, particles, and fibers with specific properties about biomedical applications, such as tissue engineering, wound dressings, and drug delivery systems, as well as into pharmaceutical applications when it is crosslinked or combined with other polymers [54,77,80,89,96,118,119,122,123,129,137,138].

The amorphous and delicate nature of sericin, however, restricts its use in the field of biomedicine [104,105,114,122]. Sericin forms’ lack of mechanical properties is one of their biggest drawbacks in many applications [54,105,114,122,139,140]. Despite attempts to improve its mechanical properties, such as adding sericin to glycerin or glycerol, these methods may negatively affect sericin’s unique properties. Understanding the relationship between preparation methods and additives is crucial for achieving optimal performance [54,114,122,132]. The molecular weight of silk proteins also influences their characteristics, with different samples produced by extraction techniques. Understanding these factors is essential for optimizing sericin’s performance [114]. Sericin can be extracted from silk cocoons through chemical treatments, enzymatic methods, and heat treatments. However, each method has limitations. Heat extraction, a safe, environmentally friendly, and cost-effective method, is chosen for *B. mori* cocoon extraction due to its chemical-free nature and lack of purification steps [120,128,141].

Sericin recovery is becoming more popular because of the substantial environmental damage that its disposal causes, as well as its advantageous characteristics. As a result, sericin’s commercialization in biomedical and pharmaceutical products may significantly influence the economy, society, and the environment [77,80,115,116,118,119,125,129].

### 4.1. Properties

**Figure 4:**
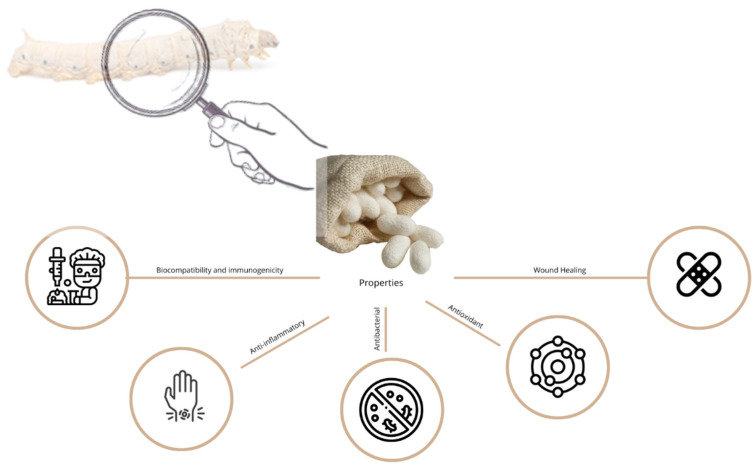
Main properties of Sericin. Figure outlining the diverse properties of Sericin: (1) Biocompatibility and immunogenicity, (2) Anti-inflammatory, (3) Antibacterial, (4) Antioxidant, and (5) Wound Healing.

Sericin demonstrates several medicinal properties that make it valuable in the pharmaceutical and medical industries [54,93,125,142]. It has been discovered to have a variety of biological (beneficial) properties, being that the most important and promising range from biodegradability, biocompatibility, immunocompatibility, Biodegradability anti-inflammatory, antibacterial, antioxidant, and photoprotective activity (Figure 4) [50,54,93,95,113,114,115,118,125,133,142,143]. Silk sericin has a large deal of potential to be employed in biopharmaceutical products due to the distinctive nature of this globular protein (which represents a family of proteins) [54,93,142].

#### 4.1.1. Biocompatibility and Immunogenicity

The capacity of a substance to carry out its intended function within the context of the intended application without adversely affecting biological systems is referred to as biocompatibility [118]. This is one of the most important considerations for materials utilized for biomedical applications [133]. Because sericin is obtained from natural sources (silkworms) and is compatible with living tissues, it is referred to as being biocompatible. Sericin exhibits little negative physiological effects when utilized in a variety of medical applications, including tissue engineering, drug delivery systems, and wound dressings. It is suitable for implantable devices and other medical applications where contact with biological tissues is required due to its biocompatibility [90,130,133].

The term “immunogenicity” describes a substance’s capacity to elicit an immunological response in the body. Sometimes, when a substance is introduced into the body, the immune system can identify it as foreign and start an immunological reaction [118]. Sericin’s immunogenicity is typically not very high. This indicates that when implanted or used in medical settings, it does not frequently cause substantial immune reactions or responses. Because it lowers the possibility of rejection or unfavorable immune reactions when sericin-based materials are utilized in the body, sericin has low immunogenicity, which is helpful for medicinal applications. This characteristic is particularly crucial in tissue engineering, where materials must easily meld with bodily tissues without triggering inflammatory or immunological reactions [133].

A study pointed out the importance of these properties when referring to conduits. The Nerve Guidance Conduit (NGC) is a hollow tube-shaped device used to treat peripheral nerve injuries. Sericin, a biocompatible and biodegradable material, has been used to create channels for nerve regeneration [133]. In another way, other studies have successfully fabricated and characterized native silk fibers, fibroin fibers, regenerated fibroin, and bionic silk. These fibers displayed good in vitro biocompatibility and no clear immunogenicity in the fibroin and sericin solution [144]. However, another one developed and characterized porous nanofibers for wound dressings, showing that nanofibers with low sericin concentrations are biocompatible and less harmful than those with high or without sericin concentrations in vitro [105].

Sericin’s biocompatibility guarantees that it works well in biological systems, and its low immunogenicity lowers the possibility of unfavorable immune reactions. These characteristics facilitate the use of sericin in a variety of medical applications while minimizing any negative effects on the body’s normal functions. However, because of the different results regarding these activities, there is still a need to continue to investigate to reach an optimized result [90,118,130,133].

#### 4.1.2. Anti-Inflammatory Properties

Although inflammation is the body’s normal response to damage or infection, excessive or persistent inflammation can result in several health problems, including chronic diseases [118]. Sericin, a natural anti-inflammatory agent, works by suppressing the expression of cyclooxygenase 2 (COX2) and NO genes, reducing Tumor Necrosis Factor-α (TNF-α) and interleukin 18 (IL-18) cytokines, and inhibiting pathways like TLR4 (toll-like receptor 4), MAK (mitogen-activated protein kinase), and NF-κB (nuclear factor kappa B), thus reducing inflammation and improving overall health [130,145,146].

Sericin’s anti-inflammatory activity was evaluated using NO production inhibition assays and 15-lipoxygenase (15-LOX) inhibitory assays. Sericin has an anti-inflammatory effect under normal conditions, but excessive NO generation can cause inflammation. In this case, sericin-phenolic conjugates were more effective in reducing NO production, indicating that grafting phenolic compounds can enhance sericin’s anti-inflammatory efficacy [147]. In another lane, it was shown that higher molecular weight sericin increased TNFα expression in RAW 264.7 macrophage cultures [101]. Also, developed composite nanofibrous scaffolds with sericin, PLGA, and ketoprofen showed favorable attachment and proliferation of human gingival fibroblasts while downregulating proinflammatory markers and upregulating IL-10 on LPS-simulated RAW 264.7 macrophages [148].

The extent and mechanisms of sericin’s anti-inflammatory effects can vary depending on aspects like its source, processing techniques, and the precise context of its application, even though it shows promise in reducing inflammation. The entire potential of sericin in anti-inflammatory treatments and formulations is still being uncovered by ongoing studies [118,130,145,146].

#### 4.1.3. Antibacterial Properties

Managing infectious diseases has become more and more difficult for healthcare systems in recent years. One of the most serious issues in wound care is the emergence of infections throughout the healing process. Microorganisms at the wound site inhibit the usual healing process from taking place, leading to additional, more serious local and systemic problems. To create biomedical products, employing biomaterials with antibacterial qualities is a promising method [89,90,114,118].

According to what has been written in the literature, cysteine, an uncharged polar amino acid, may be responsible for sericin’s antibacterial activity because of its sulfhydryl groups. These sulfhydryl groups can then interact with oxygen or nitrogen to generate weak hydrogen bonds, which results in a highly reactive chemical that influences a variety of enzymatic processes and metabolic processes in microorganisms [94,114,118].

Sericin extract at a concentration of 40 μg/mL increased osteoblast cell proliferation by 135% and showed increased antibacterial activity against *Staphylococcus aureus* (*S. aureus*). At the minimal inhibitory concentration, sericin extract significantly reduced *S. aureus* biofilm formation by up to 95% [120]. Furthermore, a binary reducing agent system made up of NaOH and NaBH_4_ was employed to produce sericin/silver nanocomposite samples using discarded sericin as a reducing and stabilizing agent. Antibacterial assays demonstrated that the samples treated with nanocomposite exhibited outstanding antibacterial activity against the bacterium *Escherichia coli* (*E. coli*) and *S. aureus* and performed better than the commercial antibacterial agent [127]. In another lane, an investigation developed a multi-functional natural substance sponge (CMC/Ser-Ag/HNT) composed of carboxymethyl chitosan (CMC), sericin-silver nanoparticle (Ser-Ag), and halloysite (HNT). Ser-Ag was synthesized in situ to enhance the antibacterial activity of the hybrid sponge against *E. coli* and *S. aureus*. Results showed that the sponge had remarkable antibacterial ability against *S. aureus* and *E. coli* [94].

This opens the door for sericin to function as a substitute functional bioactive substance, making it an important ingredient in hygiene and medical applications. Sericin is a potent antibacterial agent due to its capability to prevent the formation of biofilms and to inhibit the growth of both Gram-positive and Gram-negative bacteria. To develop powerful treatments for strains of bacteria that are resistant to antibiotics, researchers have looked into mixing sericin with other antimicrobial substances. Sericin is predicted to become an even more useful tool in the fight against microbial dangers as a result of continued research and innovation in this field [94,118,120,127,149].

#### 4.1.4. Antioxidant Properties

Although reactive oxygen species (ROS) are produced naturally by biological metabolism, they can be harmful in excessive doses. Free radicals and ROS cause a variety of disorders (cancer, cirrhosis, etc.) because they are unstable and easily react with other individuals or groups in the body [114,118,133]. Antioxidants are receiving greater attention nowadays [114]. Sericin has a powerful antioxidant activity that is widely known. Sericin’s ROS scavenging, lipid peroxidation inhibition, anti-tyrosinase, and anti-elastase actions all contribute to its antioxidant capabilities [114,118,130,133].

Natural pigments like flavonoids and carotenoids, which build up in sericin layers, can be found in *B. mori* cocoons and are pointed out as one of the factors that give sericin its antioxidant and anti-tyrosinase activity [114,118,124,130]. On the other hand, sericin’s high serine and threonine content, whose hydroxyl groups function as chelating agents for trace metals like copper and iron, is related to its antioxidant properties [118,124,130]. Moreover, since sericin can efficiently absorb ultraviolet (UV) radiation and maintain redox balance by preventing oxidative damage, it is also said to have photoprotective function. Sericin has several amino-based groups rich in hydrogen, oxygen, and nitrogen that enable substantial absorption of ultraviolet radiation with wavelengths under 200 nm [118,124,130]. Besides these properties, sericin can also lower human keratinocytes’ (HaCaT) apoptotic rates and prevent and affect oxidative stress damage brought on by extremely low temperatures in addition to UV exposure, becoming an affordable substitute for serum in cryopreservation to prevent oxidative stress damage to cells and embryos [133].

In a study that used a glucose-induced Maillard reaction, a readily crosslinked sericin film was created. The physicochemical characteristics and antioxidant functionality of the crosslinking reaction were examined. DPPH and ABTS radical scavenging tests were conducted to investigate the effect of the glucose-induced Maillard reaction on the antioxidant properties of the sericin film. The results showed that the sericin film offered UV protection, and its antioxidant activity was boosted by the glucose-induced Maillard reaction [150]. Moreover, others sought to identify the physical and chemical characteristics of a silk sericin-based poly(vinyl) alcohol (SS/PVA) hydrogel and assess both its therapeutic and toxic effects on psoriasis. SS/PVA demonstrated an antioxidant capacity through Nrf-2 as the study progressed, demonstrating that sericin has antioxidant activity [151]. Also, the covalent binding of phenolic compounds with sericin was investigated, and its effect on antioxidant activity was evaluated. Results showed that covalent modification of sericin with phenolic compounds significantly increased the antioxidative activity [147].

Sericin’s significant antioxidant action highlights its relevance in a variety of uses, from cosmetics to dietary supplements. Sericin’s status as a versatile and valuable natural substance in the areas of health, wellness, and beauty is further cemented by ongoing research into its characteristics and possible therapeutic effects [114,118,133]. However, it is also related to the cocoon strains and extraction techniques that have an impact on the antioxidant capabilities of sericin [133].

#### 4.1.5. Wound Healing

Highly biocompatible, bioactive substances that promote wound closure, degrade or metabolize at the right rate, guard against microbial infection, can be removed without causing damage to underlying tissue, and are readily available to make up the optimal wound healing treatment. Traditional methods of treating wounds, such as skin autografts, can be expensive, intrusive, ineffective, and even damaging to the wound. These ideal parameters are not currently met by any common wound treatments [76,152]. Due to their prevalence in nature, biomaterials, including collagen, elastin, and gelatin, have all been investigated as wound therapies. In the end, processing restrictions, structural deterioration, and immunogenicity in wounds are obstacles for many of these biomaterials [78,123,152].

Due to its exceptional qualities, including biocompatibility, biodegradability, water vapor and oxygen permeability, diversity in chemical functionality, and low allergenic and immunogenic profile, sericin has recently attracted significant attention for wound healing and skin regeneration [102,143,146,153,154]. Therefore, sericin is a potential biomaterial used in tissue engineering and skin regeneration, and it is widely used in biomedical sectors and the field of wound healing. For its power in promoting the healing process, it has developed a variety of sericin-based structures, like wound dressings, scaffolds, films, hydrogels, fibers, foams, spheres, capsules, and microneedles, among others [65,78,88,102,118,133,153].

Sericin’s natural ability to promote cell migration and proliferation appears to be directly tied to its ability to speed up the healing process after injury. Additionally, sericin demonstrates angiogenic action by encouraging human umbilical artery endothelial cells’ migration and tubular development [88,93,102,118,130,132,133]. Moreover, sericin can be utilized as a carrier because of its amphiphilic nature (polar side chains and hydrophobic domains), which makes it easy to bind charged medicinal molecules or hydrophobic and hydrophilic medicines [78,93,102,118,130,133]. Further, sericin can also be utilized to create drug carriers like nanoparticles and microparticles because of its chemical reactivity, which makes it easier for molecules to bind to it [78,93,102,118,130,133].

However, because of proof that it can trigger an immune response, sericin has not been widely employed as a biomaterial, but when used at the right dose, it exhibits high physiological activity. So, for sericin to be used as a biocompatible material, it is crucial to employ an optimal sericin concentration [143,146]. Furthermore, sericin’s low mechanical qualities make it necessary to combine it with other biomaterials to create scaffolds with adequate mechanical properties for bone regeneration [78,118,132,143].

In research, a heparin-based sericin hydrogel (HS) precursor solution was made through ultrasound, and fibroblast growth factor (bFGF) was added to the gel precursor solution before it gelled. The hydrogel network may continuously release bFGF when heparin is present, according to the results of the bFGF-HS in vitro release test. Animal investigations also showed that bFGF-HS can hasten wound closure, reduce inflammation, and promote vascularization when compared to negative control, HS hydrogel dressing, and bFGF. These findings imply that HS-encapsulated bFGF has extraordinary potential for tissue regeneration engineering wound repair [31]. Moreover, a sericin gel-incorporated dressing (SFD) was fabricated. The swelling capacity of the dressing (106 ± 1%) and moisture retention (≈10 h) had favorable measurements that supported its ability to maintain moisture balance, according to the results. HaCaT cells were used to verify the gel’s in vitro cytocompatibility. Finally, a mouse model with a second-degree burn was used to test the SFD’s in vivo performance. In groups that had received SFD treatment, the gross analysis and histological evaluation revealed scarless recovery. Overall, it is demonstrated that the SFD created in this work is a good contender for cutting-edge burn wound care [155]. Also, peroxidase-mediated cross-linking created an injectable fast-forming sericin-based hydrogel. After 7 days of development, in vitro tests utilizing the L929 fibroblast cell line showed that these hydrogels were noncytotoxic, encouraging cell adhesion and significant cell colonization, showing that cells maintained their viability and proliferation. Additionally, the use of a sericin-based hydrogel in an in vivo model of a diabetic lesion proved the viability of the in situ approach and showed a local anti-inflammatory impact, aiding in the healing process. Overall, it was shown that this sericin hydrogel met all of the criteria for being a novel biomaterial for wound treatment [91].

#### 4.1.6. Other Biological Activity

In addition to its well-known biological properties mentioned before, sericin exhibits a variety of other valuable characteristics. The many chemical processes that provide the energy and structural requirements of the human body are referred to as metabolic processes. Metabolic illness can develop when the metabolism changes [118]. Sericin is a compound that regulates glucose levels, insulin, and lipid secretion and prevents inflammation. It increases the expression of essential proteins like PI3K insulin receptor, phosphorylated-AKT, liver kinase, and AMPKα metabolism. Sericin also reduces the expression of TNF-α, IL-6, and P65 and inhibits nuclear factor kappa-B, making it an effective tonic for reducing blood sugar. It strengthens the PI3K/AKT signaling pathway, enhancing insulin signaling and reducing blood glucose levels. Sericin reduces blood glucose and glycosylated hemoglobin, improves glucose tolerance, and increases the mass of pancreatic beta cells, leaving an anti-diabetic effect [115,130,145]. Oral sericin can also reduce oxidative stress in the liver and pancreas, making it a potential ingredient in food or medicine to lower blood sugar [118,133,145]. Moreover, sericin’s capacity to hold onto water and unfermented fibers may also be used to treat constipation and can enhance gastric mucosa, strengthening and improving digestion [115,118].

In this lane, an analysis pointed out that sericin is a natural hormone that can help reduce triglycerides, free fatty acids, cholesterol, and phospholipids in the blood. It also helps in increasing serum adiponectin levels and interferes with cholesterol absorption by binding to bile salts. Sericin also affects body enzymes, reducing the activity of glucose 6-phosphate dehydrogenase (G6PD) and lipogenic and malic enzymes. It also suppresses plasma glucose and insulin, improving lipid and carbohydrate metabolism and preventing obesity and weight gain. However, sericin cannot completely address the physiological changes due to obesity. Oral sericin plays a crucial role in inhibiting lipogenesis and regulating adiponectin production, effectively preventing the increase in blood fat and obesity caused by food. Ultimately, sericin can help reduce obesity and improve overall physical condition [145].

By preventing cell glycolysis and lowering lactate generation, sericin exerts antiviral effects that promote IFN activation and antiviral responses. Sericin enhances RIG-1-MAVS signaling to trigger IFN expression, which aids in cell innate immune response to RNA virus infection. F5-SP, a peptide produced from sericin, operates similarly to sericin in the treatment of viral infection. Sericin and F5-SP peptides have been shown in vivo experiments to reduce viral pathogenesis and increase the survival of mice with RNA virus-induced respiratory tract infections, demonstrating their potential for use in the prevention and treatment of viral infectious disorders [80,130,133].

Numerous cognitive benefits of silk, including antidepressant, anxiolytic, and memory-improving properties, have been noted in traditional medicine. The effects of silk on neurodegenerative diseases like Alzheimer’s and Parkinson’s have been shown in numerous research. Increased ROS and oxidative damage are frequently linked to a variety of brain illnesses, such as depression, neurodegeneration, aging, memory loss, and other disorders of the brain. According to studies, sericin can keep the acetylcholine (Ach) levels in the brain’s cholinergic regions stable. Sericin accomplishes this by inhibiting Acetylcholinesterase (AChE) action and lowering its concentration. Sericin is regarded as a cognitive enhancer appropriate for Alzheimer’s disease because it often produces neuroprotection on the cholinergic system. Additionally, Sericin improves memory impairment in Alzheimer’s patients, making it well-known as a supplement to improve cognitive abilities. As an antioxidant, sericin can, therefore, help treat Alzheimer’s disease [115,130,145].

The anticancer effect mentioned for sericin is associated with several types of cancers, and it is related to the following mechanism: sericin raises intracellular ROS, prevents the cell from progressing past the G1 phase, and kills the cell. Sericin, therefore, inhibits the growth of cancer via having a pro-oxidative impact [145]. Sericin can thereby prevent the growth of colorectal tumors and the division of lung cancer cells. There are additional reports of sericin’s ability to activate natural killer cells, which are renowned for their capacity to recognize malignant or contaminated cells, as well as to boost their maturation and cytolytic activity. Sericin microspheres or nanoparticles could be created for immunotherapy purposes because, upon regeneration, they would reveal hydrophobic regions beneficial for macrophage activation, improved phagocytic ability, and stimulation of proinflammatory cytokine production [115,137,145].

### 4.2. Applications

In the silk industry, sericin was viewed as waste. However, its qualities have been discovered today, and it is employed in various industries, including biotechnology, pharmaceuticals, cosmetics, food, and textile. According to recent research, some of the examples pointed out are that sericin can be used as sunscreen and to create UV protection in textiles since it absorbs ultraviolet radiation. Surgical sutures, gloves, pads and bandages, aprons, and sheets are all made with sericin, a substance with antibacterial qualities. In toothpaste and shaving cream, it can be used to stop the spread of HIV [113,114,118,145].

In the medical and pharmaceutical industries, drug delivery methods based on sericin are gaining popularity as potential replacements for polymers, nanoparticles, and other pharmaceutical carriers. Sericin is employed in several medicinal applications, such as formulation stabilization, diffusion modification, and solubility enhancement. Additionally, an antioxidant and anticoagulant, it has health advantages such as constipation alleviation, colon cancer development inhibition, and improved mineral absorption. Sericin’s high porosity, enhanced cell cohesion, and vitality make it ideal for drug delivery, bonding, matrices, scaffolds, and films. It is also utilized in artificial skin, prosthetics, blood vessels, contact lenses, and wound dressing. Sericin has the potential to be used as a wound-covering material due to its sulphonation-induced antithrombotic, antibacterial, collagen-producing, hydrophilic, and biocompatibility qualities [90,114,118]. However, it was pointed out that its limitations were due to its extraction method, high solubility in water, and animal origin. It cannot be used as drug delivery systems or tissue engineering scaffolds alone due to its low mechanical properties. Therefore, sericin-based scaffolds are typically prepared in combination with other components [118].

Regarding the cosmetics industry, sericin, with its biocompatibility, biodegradability, and wettability, is used in skin, hair, and nail cosmetics for its skin elasticity, anti-wrinkle, and anti-aging benefits. Moisturizers containing wetting agents, like vegetable glycerin, water, jojoba oil, vitamin E oil, and sorbitol, are developed to prevent skin dehydration. Sericin gels with hydroxyproline increase the epidermal layer and reduce skin impedance, while lotions with 1% sericin and 4% D-glucose have moisturizing and conditioning effects. Sericin also improves UV filter light screening in sunscreen compositions and absorbs sweat and fat from the skin’s sebaceous glands [114]. Plus, due to its unique physical-chemical makeup, sericin has also been mentioned as a component of skin and hair care cosmetic formulations. For instance, it has been shown that silk sericin has antioxidant and tyrosinase inhibition activity, indicating its potential for whitening, which could be advantageous for cosmetic applications. It has also been shown that a hair care product based on silk proteins (fibroin, sericin, and other proteins) has protective effects against the harm brought on by hair bleaching and coloring [118]. Today, sericin is already used as an ingredient as a moisture agent in some cosmetic products such as Temt^®^, Revital^®^, and Kristida^®^ [111].

In the food industry, the FDA has listed sericin and its derivatives as authorized additives (GRAS). Sericin aids in fermenting fortified foods and dietary supplements, increasing mineral absorption and antioxidant activity and reducing cholesterol, triglycerides, and liver fat accumulation. It also protects fruits and vegetables from aging and can be used in bread baking for improved digestion, uniformity, and easy swallowing. Sericin’s antioxidant activity and emulsifying activity make it a promising ingredient in salad dressing [114,141]. However, additional research is necessary to assess its use as a functional food additive under the purview of regulatory authorities. Last but not least, as sericin is a by-product of the production of silk, this protein may be valuable from an economic and environmental standpoint and have prospective uses in food [141].

In the textile industry, sericin can modify the surface of fibers and fabrics by coating cellulose fibers and wool. It reduces free formaldehyde content, increases resistance to electricity, skin irritation, and allergic reactions, and enhances antibacterial ability. Sericin can overcome polyester’s hydrophobicity and UV absorption properties and enhances antistatic and radical scavenging behavior. It can also be combined with other materials to provide antimicrobial properties in nanofibers for wound dressing. Sericin-coated fabrics are used in diapers, diaper liners, and medical textiles for wound dressings and healing abrasive skin injuries. Studies are exploring sericin’s potential for textile substrate finishing [114,118].

Sericin’s numerous uses essentially illustrate how important it is for advancing sustainability, hygiene, and good health. Research’s potential in both established and developing businesses is anticipated to increase as it develops, opening up new applications and enhancing the industries it supports. Sericin is a prime example of the inventiveness of utilizing natural resources in many significant ways to meet various demands in our contemporary environment [114,141,145].

### 4.3. Future Prospects

The development of ecologically friendly methods for material processing and fabrication has received increased attention in recent years as a result of rising environmental concerns. In some circumstances, notably in the biomaterials industry, this might also entail actions to substitute safer alternatives for materials that can have negative impacts on human populations [18,150,156].

Sericin, a byproduct of the silk industry, is beginning to show significant promise in biomedical research and applications thanks to recent developments and the creation of sericin-based biomaterials for tissue engineering and regenerative therapy. Pertinent advancements may alter the silk industry’s existing situation and advance tissue engineering and regenerative medicine, as well as the modernization and transformation of traditional industries. Although several silk sericin-derived biomaterials (ranging in size from nano to bulk) have been created and studied, there have never been any sericin-derived medicinal goods on the market [18,133].

Research on the creation of sericin biomaterials has grown recently as awareness of sericin’s diverse properties has grown [150,157,158]. Sericin has a range of uses, from treating injuries to decorating sustainable designs. However, its characteristics change depending on the environment, which leads to partial withdrawal or suspension of studies [120,130,150,157,158,159].

Despite its beneficial properties, several points need to be addressed. Sericin´s bioactivity and properties need to be understood. The interaction between inorganic and organic phases, as well as the degradation mechanisms of different forms of sericin, must be investigated [119]. Sericin’s bioactivities can vary greatly, and the extraction method may also affect its bioactivity [130]. Currently, there is no method to extract a specific type of sericin alone, and their biological functions and activities differ across studies. Therefore, effective anti-sericin antibodies are needed to analyze the main sericin types in different molecular weight ranges [160]. Improvements in medical silk cocoon production include mechanization and automation of sericulture, development of chemically stable raw fibroin material, understanding of the relationship between processing and properties, and development of storage protocols for long-storable and stable biopolymers [130]. Additionally, systematic studies on dimensional stability and aging of silk products can improve the quality and stability of medical devices and materials [18,157]. Overall, the potential of sericin in biomedicine is vast, but further research is needed to fully understand its potential [18,130,157].

When it comes to the development of medical devices, the main issue is the lack of standardization, affecting the entire production chain from silkworm breeding to the final medicinal device [157]. According to the FDA’s classification management regulations for medical devices, new devices must show that they are substantially equivalent to predicate devices in terms of their efficacy and safety. Products made from sericin, however, have problems with biosafety, effectiveness, mechanical properties, and batch variations. Exploring the bioactivities of sericin and its derived peptides, identifying peptide sequences and conformation, conducting preclinical and clinical studies on biosafety and efficacy, and improving the mechanical properties of sericin-based materials should be the main areas of research on sericin to address these problems [133].

Overall, despite certain “uncultivated lands” in the study of sericin, this naturally occurring protein shows significant promise for the development of innovative materials for tissue engineering and regenerative therapy [133]. In general, developments in material science, engineering, and basic science with sericin offer great promise for the future. The commercialization of items made from silk may result from research on silk-based materials [158].

## 5. *Chelidonium majus* L.

Herbal therapy is regarded as a successful substitute for pharmaceutical medicine. Many successful cures are derived from raw components found in the wild and introduced medicinal plants. Currently, active herbal components comprise roughly 40% of medications, and the demand for medical herbs is only growing [161].

Pharmacological and biochemical investigations of metabolites belonging to the Papaveraceae family have provided insights into their possible applications in several medical fields in recent years. Due to their capacity to yield several alkaloids with notable bioactivity, Papaveraceae, the poppy family of flowering plants, are highly interesting in the context of their potential medical applications [106,162].

One valuable medicinal plant of the Papaveraceae family is the Greater celandine, *Chelidonium majus* L. [161,163,164]. This perennial herbal plant is widely distributed in regions of moderate climate on the continents of Europe, Asia, and North America [108,165,166]. The body is 50–100 cm tall, branching, and covered in a scattering of feathers. The leaves are slit along a single stalk and stacked one above the other. The root’s side leaves are thicker and arranged in a lengthy leaf band. There are no bands grouped on the upper leaves of the stem [108,165]. Greater celandine flowers are yellow, four-petaled, and radially arranged in loose corymbs. The flowering phase’s phenological period runs from May to September. The fruits resemble long, cylindrical capsules when they are formed. The plant has a milky sap that is yellow-orange in hue throughout. Although cross-pollination is also possible, the flowers self-pollinate. The seeds should ideally be sown in the fall for optimal cultivation [165].

*C. majus* has been utilized in traditional medicine for generations due to its abundance of several biologically active components, such as flavonoids, phenolic acids, and alkaloids [108,109,161,163,166]. Being one of the oldest medicinal plants, it has been used to treat a variety of illnesses since ancient times [106,109,167,168].

### 5.1. Chemical Characteristics

One of the most widely used and accessible methods for estimating the intraspecific diversity of plants is bio-morphological characterization. Each genotype of a plant has distinct characteristics, such as plant height, leaf length and width, flower and fruit size and shape, trichome morphology, and leaf, seed, and fruit color. These characteristics are used to identify species, cultivars, or hybrids and confirm their homogeneity and stability. Moreover, the chemical identification and assessment of the plants is particularly significant, especially for therapeutic purposes [106,161,169].

Due to its extensive usage in both formal phytotherapy and folk medicine, *C. majus* is a plant of significant interest. It is known that this plant produces a wide variety of secondary metabolites, which give it its medicinal qualities [107,161,164,170]. More than just the potent bioactive alkaloid components (coptisine, chelidonine, chelerythrine, sanguinarine, berberine, protropine, dihydrocoptisine, stylopine, and isochelidonine), greater celandine also includes flavonoids, resin, fruit acids (malic, citric, and tartaric), vitamin C, volatile oil, and metals [109,161,164,166,171,172,173,174,175]. Current pharmacological research has focused particularly on the plant’s several isoquinoline alkaloids [164,167,168,174,176,177].

The plant’s aerial parts are made up of isoquinoline alkaloids, which include benzophenanthridine (chelidonine, chelerythrine, sanguinaria), protoberberine (coptisine, stilopine, berberine), and protopine (protopine, alocriptopine) [165,169,178].

Carotenoids [173,178]; flavonoids [165,173,174,178] like quercetin [175]; organic acids [178] like malic and citric acid [173]; phenolic acids [164] like gentisic acid, hydroxybenzoic acid, and hydroxycinnamic acid [173], transcaffeic acid and chlorogenic acid [175]; and proteins [178].

The amount of alkaloids, tannins, and polyphenols is higher in the rhizome. Cu, Fe, Mn, Cr, and Zn are among the metal ions found in the plant’s extracts, which support the intended therapeutic benefits [164,174]. Furthermore, it possesses antioxidant properties that are connected to polyphenolic substances [174]. The plant’s roots, stems, leaves, flowers, and pods are among the sections that contain varying amounts of yellow latex [173]. Latex extracts are the most appropriate method for producing preparations high in alkaloids [174], which are widely used to treat eczema, corn, fungal infections, and skin tumors [171].

Despite the toxicity and mutagenicity of some compounds, this plant is still extensively studied due to the possibility of synthesis of derivates with reduced toxicity. Determining the compound’s effect and mechanisms of action towards various activities, as well as the range of useful concentrations, and testing mixtures of chosen compounds in different proportions to one another would allow exploring mechanisms of potential synergy (the addition or abolition of biological effects) are particularly important research directions for this aspect [106,177,179].

The *C. majus* plant demonstrates a wide spectrum of bioactive/pharmacological qualities and therapeutic effects as a result of the numerous bioactive substances mentioned above [164,171,178,180].

### 5.2. Properties

Research is still being done on the bioactive components of medicinal plants and their multifunctional properties, which include immune modulation, health benefits, and personalized treatment. Natural compounds, especially secondary metabolites like flavonoids, terpenoids, alkaloids, and carotenoids, which are necessary for medicinal drugs, functional meals, and cosmetics, can be synthesized more easily in plants thanks to their metabolic pathways. It is essential to comprehend this metabolic variability and bioactivity to create novel therapies and functional food [173].

**Figure 5:**
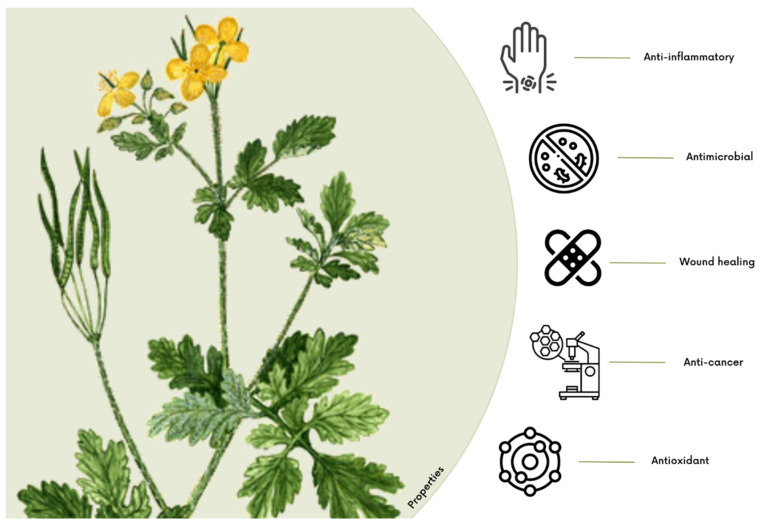
Main properties of *C. majus*. Figure outlining the diverse properties of *C. majus*: (1) Anti-inflammatory, (2) Antimicrobial, (3) Wound healing, and (4) Other properties.

*C. majus* is one of the oldest medicinal species, having been in use since ancient times [106]. As mentioned before, this plant is known for its crucial alkaloid and flavonoid content that checks its many pharmacological properties (Figure 5) [84,106,108,109,161,173]. Despite its approval by the European Pharmacopoeia, studies have pointed out its toxicity because of its compounds. According to the European Medicines Agency (EMA), the toxicity of dried parts of *Chelidonium majus* L. in a normal dose is low, but severe and irreversible hepatotoxicity can happen at a high dose or with chronic uptake. So, further investigation is needed to determine possible toxic effects in daily administration and terms of use. To prevent any unintended injury, it is crucial to carry out morphological and anatomical research to produce trustworthy diagnostic features for the identification of commercial samples and raw materials [106].

#### 5.2.1. Anti-Inflammatory Properties

As mentioned before, inflammation is the body’s initial defense response, protecting cells and tissues from microbial invasion, antigen entry, and injury [118]. Medicinal plants, which produce secondary metabolites with medicinal effects, are used for treatment and drug development, and greater celandine is no exception. This plant has been studied for its anti-inflammatory properties, which are attributed to the presence of various bioactive compounds [179,180,181].

The alkaloids present in *C. majus*, isoquinoline alkaloids, sanguinarine, and chelerythrine, have anti-inflammatory functions [179]; these are potent inhibitors of inflammation-mediating enzymes such as 5-lipoxygenase [181]. These compounds are pharmacologically active with a strong anti-inflammatory effect and potential therapeutic use [179,181]. *C. majus* is one of the natural sources of berberine and other compounds derived from protoberberine, described as potent anti-inflammatories [182].

A MeOH extract of *C. majus* aerial parts was tested to see if it could decrease the generation of nitric oxide (NO) in RAW 264.7 macrophages when lipopolysaccharide (LPS) was added (IC_50_ value: 31.5 μg/mL). The findings validated the application of *C. majus* as a medicine for the treatment of inflammatory illnesses and the role that isoquinoline alkaloids play in this plant’s anti-inflammatory properties. They concluded that treating inflammatory illnesses brought on by an overabundance of NO may benefit from using the aerial portions [180]. In the same way, the anti-inflammatory activity of isolated compounds of *C. majus* was investigated. Results showed that the alkaloids in the plant, such as chelidonine and 8-hydroxydihydrosanguinarine had strong inhibitory activity to RAW264.7 cells. In particular, sanguinarine and chelerythrine, two of the main alkaloids in *C. majus*, showed potent inhibition of 5-lipoxygenase in polymorphonuclear leukocytes with IC_50_ values of 0.4 and 0.8 μM, respectively [181]. Also, the effects of *C. majus* root extract on IL-1β, IL-8, and TNF-α secretion in human polymorphonuclear leukocytes were studied. Results showed that berberine, chelidonine, and chelerythrine decreased TNF-α secretion, while sanguinarine was the most potent inhibitor. Coptisine (extract 1.25–12.5 μg/mL) was highly cytotoxic and slightly decreased cytokine secretion. They also noted that the alkaloids were active at very low concentrations (0.625–2.5 μM), but their potential cytotoxic effects, except for chelidonine and chelerythrine, should not be ignored [179].

So, it is important to note that while these findings suggest the potential anti-inflammatory properties of *Chelidonium majus* L., more research is needed to fully understand the mechanisms and establish its efficacy and safety [179,180,181].

#### 5.2.2. Antimicrobial Activity

Among its various biological activities, the antimicrobial potential of greater celandine has garnered significant attention in recent research endeavors. Studies have revealed that this plant possesses antimicrobial activity against a wide spectrum of microorganisms, including bacteria, viruses, and fungi [106,167,183]. The plant’s extracts show antimicrobial activity due to their alkaloid composition [169]. These compounds have been found to inhibit the growth and proliferation of various bacterial strains, making *Chelidonium majus* L. a potential source of natural antimicrobial agents [106,167,183].

The isoquinoline alkaloids, chelerythrine, sanguinarine, and chelidonine, are the most effective in antimicrobial activity [74]. These alkaloids showed strong antibacterial activity against various bacteria (Gram-positive and Gram-negative), such as *Staphylococcus aureus* (*S. aureus*) and *Pseudomonas aeruginosa* (*P. aeruginosa*), and fungi, such as *Candida albicans* (*C. albicans*), very similar to the antimicrobials used on the market, such as alcohol gel and antibacterial soaps. Specifically, chelidonine has strong antifungal activity [74]. Protoberberine derivatives, such as berberine, are pharmacologically active with excellent antibacterial properties [182]. The latex of *C. majus* is used to treat warts due to its strong antiviral effect [109,174,184].

The antimicrobial activity of seven alkaloids and *C. majus* extracts from plants derived from natural habitats and in vitro cultures was evaluated. The results have shown that all tested plant extracts manifested antimicrobial activity related to different chemical structures of the alkaloids. Of the seven individually tested alkaloids, chelerythrine was the most effective against *P. aeruginosa* (MIC at 1.9 mg/L), while sanguinarine against *S. aureus* (MIC at 1.9 mg/L). Strong antifungal activity was observed against *C. albicans* when chelerythrine, chelidonine, and aerial parts extract were used [169]. Moreover, the antibacterial activity of extracts from selected plants of the Papaveraceae family against planktonic and biofilm forms of the multidrug-resistant clinical strain of *Helicobacter pylori* (*H. pylori*) was determined using a broad spectrum of analytical in vitro methods. The results showed a potent antimicrobial activity of extracts from *C. majus* alone and in combination with tested synthetic substances (amoxicillin, 3-bromopyruvate, or sertraline) against planktonic and biofilm forms of *H. pylori* [162]. In another lane, the modes of action of three alkaloids isolated from *Chelidonium majus* L. against tobacco mosaic virus (TMV) were investigated. The results demonstrated that chelerythrine is an excellent botanical anti-TMV factor, and chelidonine is a potential plant resistance elicitor [167].

It is important to note that while greater celandine shows promise in terms of antimicrobial activity, the use of herbal remedies should be approached with caution. Proper preparation, dosage, and consultation are essential to ensure both safety and effectiveness. Additionally, as research in this area is ongoing, more studies are needed to fully understand the mechanisms and potential applications of greater celandine’s antimicrobial properties [162,167,169,185].

#### 5.2.3. Wound Healing

Skin disorders like dermatitis, eczema, psoriasis, wounds, and burns often require antibiotics. However, plant-based medications, dietary supplements, and cosmetic products are gaining popularity for milder symptoms [172]. Plant components have anti-inflammatory, antibacterial, and immunostimulant properties, and there is promise in using plant extracts to heal wounds [186]. New research on skin microbiota’s role in skin homeostasis may help better understand the effects of plant-based treatments [172].

*C. majus* has several bioactive components that are responsible for the plant’s healing power. Isoquinoline alkaloids, such as sanguinaria and berberine, act in wound healing due to their pharmacological properties, such as being anti-inflammatory and antimicrobial [177].

The vulnerability of botanical medicines made from local raw materials in the treatment of sturgeon bacterial pathologies in a controlled environment was explored. By assessing the degree of wound healing, the results showed that *Chelidonium majus* L. infusion reduced the surface area of wounds better than other materials used and the control [186]. In another lane, it was used for the first time a needleless emulsion electrospinning technique for incorporating *Chelidonium majus* L. in Polycaprolactone (PCL)/Polyvinyl Alcohol (PVA)_Pectin (PEC) nanofibrous meshes. The results obtained revealed that the produced PCL/PVA_PEC nanofibrous meshes containing *C. majus* extract displayed morphological characteristics similar to the natural extracellular matrix of the skin (ECM), and the produced meshes showed beneficial properties to support the healing process [84]. Moreover, another reported case involved a four-year-old boy who followed a treatment using a milky sap of celandine, and the hand wart was cured [165].

Plants have been used to treat skin diseases since ancient times, but their mechanisms remain unclear [172,186]. *C. majus* exhibits potential wound-healing properties due to its anti-inflammatory, antimicrobial, cell-proliferative, analgesic, and angiogenic effects [165,172,186]. However, one of the major problems is the lack of well-described and reliable models that can be used for the investigation of the interactions between the skin microbiome and the plant extracts [172]. Responsible usage and further scientific research are necessary to harness its benefits effectively in wound care [165,172].

#### 5.2.4. Other Properties

*Chelidonium majus* L. is a well-known medicinal herb and is widely used against various diseases. Besides the properties already mentioned, this plant exhibits a variety of other valuable characteristics [107,187,188].

*C. majus* is known for its anti-cancer properties, as it has been used since 1996 to treat various cancers, such as skin cancer [187]. The bioactive compounds responsible for its anti-cancer properties are berberine [166,177], sanguinaria [177,189], chelidonin, and chelerythrine [171]. Several studies have been carried out on bioactive compounds with anti-cancer properties, the most potent being berberine [177,182,189] and sanguinarine [171]. Berberine is a quaternary plant isoquinoline alkaloid and has several therapeutic and biologically important properties [182]. It inhibits cell proliferation in various cancers and induces apoptosis in human cancer cells (breast, pancreatic, cervical, and prostate) [182,189]. This alkaloid is non-toxic to normal cells and cytotoxic to cancer cells [177]. Sanguinaria is another benzophenanthridine-type alkaloid that has several pharmacological activities, including anticancer. It inhibits the cell cycle and induces apoptosis in various types of cells at cell surface receptors belonging to the tumor necrosis factor (DR5) receptor family and in mitochondrial pathways. It is toxic to normal cells and cytotoxic to cancer cells [177].

A study evaluated the cytotoxicity and proapoptotic activity of sanguinarine, berberine, and extracts of *Chelidonium majus* L. Results showed Sanguinarine and *C. majus* extract exhibit significant cytotoxicity against all studied cell lines. Lower cytotoxic activity was demonstrated for berberine. Furthermore, berberine, sanguinarine, and *C. majus* extract altered the expression of apoptosis-related genes in all tested cell lines, indicating the induction of apoptosis [177]. In the same lane, another isolated the major latex proteins (MLP) from *C. majus* latex, identified accompanying low-molecular compounds, and analyzed their joint cytotoxic activities against cervical cancer cell lines. The results showed a decreased in vitro viability of human cervical cancer cells (HPV-negative and HPV-positive) [166].

Also, the cytotoxic effect of *Chelidonium majus* L. extracts obtained from different parts of the plant collected at various vegetation stages was investigated. The highest cytotoxic activity against melanoma A375 cells was observed for the *C. majus* root extract, with an IC_50_ of 12.65 μg/mL. The same extract was the most cytotoxic against SK-MEL-3 cells (IC_50_ = 1.93 μg/mL), while the highest cytotoxic activity against G361 cells was observed after exposure to the extract obtained from the herb of the plant. The cytotoxic activity of the plant extracts against melanoma cells was compared with the cytotoxicity of the following anticancer drugs: etoposide, cisplatin, and hydroxyurea. In most cases, the IC_50_ values obtained for the anticancer drugs were higher than those obtained for the extracts. The most cytotoxic extract obtained from the root of *Chelidonium majus* L. was selected for in vivo cytotoxic activity investigations using a *Danio rerio* larvae xenograft model. The results obtained in this model showed a great reduction in the number of cancer cells in the *Danio rerio* organisms after their exposure to the extract. The effect was similar to that obtained after treatment with etoposide [189].

The global pandemic of obesity has raised serious concerns about public health and economic hardship. A common factor in obesity and type 2 diabetes that affects the skeletal muscles, liver, and pancreatic beta cells is insulin resistance. The active ingredient in *C. majus*, berberine, has been studied before on several metabolic pathways [168]. According to the same authors, this plant has been studied on several metabolic pathways in the past. Additionally, it has been studied concerning AMP-activated protein kinase pathway activation in swine adipocytes. Moreover, it works by preventing 3T3-L1 adipocytes from phosphodiesterase, which attenuates cAMP-induced lipolysis. By regulating AMPK activity and biological activities like inhibiting cholesterol synthesis, lipogenesis, and adipocyte lipolysis, the phytocompound from *C. majus* also improves lipid metabolism and glucogenesis in liver disorders. It also regulates glucose metabolism by lowering insulin resistance and raising insulin secretion.

Moreover, researchers are increasingly exploring natural sources of molecules with biological potentials, such as polyphenols from plants, to protect against oxidative stress, a major cause of many diseases [107]. The antioxidant activity of root and stem extracts from *Chelidonium majus* L. was assessed using oxidative stress biomarkers and antioxidant enzyme activity in equine plasma. The extracts from *C. majus* roots and stems showed effective antioxidant activity when incubated with equine plasma. They improved antioxidant defenses and increased total antioxidant capacity, suppressing oxidatively modified proteins [107]. In the same lane, another investigated the different plant parts like the leaf, stem, flower, pod, and root of *C. majus* using spectroscopic and chromatographic methods. The flower extract demonstrated the highest antioxidant activity (46.62%), outperforming other plant parts in various antioxidant assays, including ABTS, DPPH, and phosphomolybdenum assays [173].

In another type of study, a case series of 20 outpatients treated with greater celandine was described and showed significant clinical improvement within a few days. The patients had mild symptoms, and the treatment was well-tolerated. The study suggests that *Chelidonium majus* L. may be a promising healing treatment for COVID-19 [185].

### 5.3. Future Prospects

These days, one of the most popular research specialties is drug development. Instead of creating new synthetic pharmaceuticals, scientists are currently concentrating on discovering unique drugs from natural sources. Historically, people have frequently utilized plants for medicinal purposes. Thanks to advancements in contemporary medicine, we are now able to identify new mechanisms of action and explain existing ones [163,176].

Plant extracts are extremely intricate blends of several substances that may have antagonistic or synergistic effects on biological function [177]. In this area, natural substances are extensively researched, and significant strides have been achieved in figuring out the molecules’ chemical structures and modes of action. Alkaloids, flavonoids, lignans, saponins, terpenes, or taxanes are some of the substances that are important in treatments [176].

A growing trend in response to the possible adverse effects of synthetic medications is the demand for complementary therapies, which include herbal medicine [165]. Belonging to the Papaveraceae family, the greater celandine is one of the most significant herbs in traditional medicine. *C. majus* possesses antioxidant, anticancer, antithrombotic, immunomodulatory, anti-inflammatory, antibacterial, and antiviral properties, as demonstrated by several investigations. The plant’s several alkaloids, including carotene, flavonoids, vitamin C, phenolic acid, coptisine, chelidonine, homochelidonine, and berberine, are responsible for these varied effects [106,163,171,183,186]. The plant’s aerial portions, leaves, and flowers are used both externally and orally. However, the latex is mainly applied externally to cure skin conditions. The most popular therapeutic application of the plant is the direct application of latex to eliminate warts [106].

The wide range of chemicals included in plant extracts can function in concert to produce a more potent effect than any one of the compounds alone, which is one of the main benefits of utilizing them as a whole [163]. When compared to conventional techniques (such as antibiotics and other chemotherapies), botanical therapy enables more efficient and timely medication delivery than normal medicines, without the negative side effects [186].

Like other wild plants, greater celandine has intriguing qualities that could make it profitable [171]. However, there is a need for further investigation of the mechanism of action of *C. majus* [165,176], the determination of elemental content [164,171], the determining of the seeds morphological characteristics in the fruits and grains composition formed during plant ontogeny [108,171], the influence of cultivation practices [171], and the isolation of the active ingredients [165], taking into account several variables, including production procedures, manner of storage, the safety of the long-term use of the extract [163]; the different extractions methods, variety of chromatographic analyses and variations in the different plant parts [173], dose and the duration of application [163].

## 6. Conclusions

Wound healing is a complex process involving cell growth, re-epithelialization, collagen fiber deposition, and tissue regeneration [2,5,23,65]. Selecting suitable polymers, bioactive compounds, and wound dressings is crucial for enhancing the healing process. There is no single dressing for all wound types, so the future challenge is developing smart dressings with antimicrobial, anti-inflammatory, and antioxidant properties [24,44,65]. Among other reasons, people are becoming more interested in using medicinal herbs because they are less expensive and have fewer negative effects than commercial goods. Nonetheless, using medicinal plants to treat wounds is still in its early stages and is a holistic approach [44,65].

For hundreds of years, wound dressings have changed and evolved. Although there are ancient records of dressings dating back thousands of years, the traditional understanding of wound dressings did not emerge until the last several centuries [27,33,70]. With more than 3000 distinct wound dressings on the market, healthcare professionals can select the best dressing by taking into account every aspect of wound treatment. Although there is a wide range of options, no one type of wound dressing is inherently better than another, and several types of dressings are frequently beneficial for different types of wounds [27,33,39,44]. In the last ten years, a new class of wound dressings has been launched because of technological advancements. Modern material technologies are used in smart dressings to help with common non-healing wounds [27,33,70].

In the next 5–10 years, the wound care field is set for a significant transformation, fueled by breakthroughs in biomaterials, regenerative medicine, and personalized healthcare. As our knowledge of the molecular and cellular processes involved in wound healing expands, we can expect the emergence of advanced dressings that do more than just protect wounds—they will actively facilitate tissue regeneration and accelerate healing. The combination of natural substances like Sericin and Chelidonium majus with state-of-the-art technologies such as nanofibers, hydrogels, and smart dressings with real-time monitoring capabilities is likely to become increasingly common. Additionally, the focus on sustainable and biocompatible materials will inspire innovations in eco-friendly wound care solutions that not only improve patient outcomes but also reduce environmental impact. Personalized wound care, tailored to the unique genetic, microbial, and wound-specific characteristics of each patient, could become the norm, leading to more precise and effective treatments. In this rapidly evolving landscape, interdisciplinary collaboration will be crucial as advances in materials science, biology, and technology come together to create the next generation of wound care products that are both innovative and widely accessible.

To sum up, this review emphasizes the promising therapeutic qualities of Sericin and *Chelidonium majus* L. as possible wound dressings. The silk extract known as sericin has strong antibacterial and anti-inflammatory qualities, which help it heal wounds effectively [114]. Plant material *C. majus* has the potential to reduce inflammation and promote tissue regeneration [108]. These two components working together in wound dressings have a lot of potential to improve the overall healing process.

The study also emphasizes how crucial it is to investigate natural and alternative materials for wound care because of their biocompatibility and ability to hasten the healing process. The creation of sophisticated and long-lasting wound dressings that have the potential to completely transform current wound care procedures is clearly on the horizon as it will continue to explore the processes underlying the therapeutic actions of Sericin and *Chelidonium majus* L [2,7,10,11,30,67,190].

While numerous studies in the field have focused on evaluating various dressings, often concentrating on synthetic or single-component systems, this research takes a novel approach by exploring the synergistic effects of combining Sericin and *Chelidonium majus* L. Unlike traditional studies that typically emphasize the benefits of individual materials, this paper highlights the enhanced therapeutic potential that emerges from the interaction between these natural substances. By demonstrating how their combined use can lead to superior wound healing outcomes, this work not only deepens our understanding of effective dressing formulations but also lays the groundwork for developing more sustainable, biocompatible, and effective solutions. Compared to other contributions, this study provides a forward-looking perspective, setting the stage for future innovations in wound care.

However, to verify the safety and effectiveness of these materials in various clinical situations, more research and clinical trials are necessary [11,114]. For practical implementation, the production’s cost-effectiveness and scalability also need to be taken into account [11,36]. These natural dressing materials could be essential in the future for encouraging a more effective and comprehensive approach to wound care, which would eventually improve patient outcomes and lessen the strain on healthcare systems [2,10,11,36,114].

## Figures and Tables

**Table 1:** Factors that influence the wound healing process. Table summarizing factors and their influence on wound healing: systemic factors (age, chronic illnesses, genetic components, stress, mental state, smoking, and nutrition) and local factors (temperature, blood supply, oxygenation, and infection).

Factors		Description	References
Local Factors	Temperature	Maintaining an appropriate body temperature promotes enzymatic reactions and cellular activities involved in healing	[13,31,49]
	Blood Supply	Proper blood circulation delivers oxygen, nutrients, and immune cells needed for healing. Poor circulation hinders healing	[11]
	Oxygenation	Adequate oxygen supply is crucial for cellular activities and collagen synthesis	[11,31]
	Infection	Infections delay healing by increasing inflammation and impeding tissue repair	[11,31,43]
Systemic Factors	Age	Younger individuals tend to heal faster due to more robust cell activity and collagen synthesis	[11,31]
	Chronic illnesses	Conditions like diabetes and immune disorders can impair wound healing by affecting blood flow and immune responses	[11,13,31]
	Genetic components	Several genetic factors affect wound healing; for example, men have different inflammatory responses and take longer to heal acute dermal wounds than women	[31]
	Stress and Mental State	Stress can hinder immune responses and delay wound healing. Positive mental states can support healing	
	Smoking	Smoking reduces blood flow, delays wound healing, and increases infection risk.	
	Nutrition	Adequate intake of nutrients, especially protein, vitamins (C, A, E), and minerals, is essential for cell growth and tissue repair	[11,31]

**Table 2:** Sericin and *Chelidonium majus* L. dressing versus other wound care drug delivery systems. Comparative table that contrasts the effectiveness and safety of Sericin and *C. majus* dressing with other common drug delivery systems used in wound care.

	Sericin & *Chelidonium majus* L.	Hydrocolloid	Silver-Based	Alginate	Topical Antibiotics	References
Wound healing	Promotes tissue regeneration, reduces inflammation, and accelerates healing due to synergistic natural components	Maintains a moist environment, promotes autolytic debridement, and moderates healing properties	Effective in reducing microbial load, especially in infected wounds, but may slow healing due to cytotoxicity	Highly absorbent, effective for exudative wounds; promotes a moist environment for healing	Prevents bacterial infection but may not actively promote tissue regeneration; risk of resistance	[73,74,75,76,77,78,85,92,94,103,111,112]
Anti-inflammatory property	Strong anti-inflammatory effects from *C. majus*, reducing wound site inflammation.	Minimal to no anti-inflammatory properties.	Indirect anti-inflammatory effects through reduction of microbial load.	Low to no anti-inflammatory properties.	Anti-inflammatory due to infection control but not directly through tissue effects.	
Anti-microbial property	Moderate antimicrobial properties; *C. majus* contributes to reducing bacterial growth	Low to none; relies on a sealed environment to limit infection	High antimicrobial efficacy against a broad spectrum of bacteria	Low to moderate; relies on the environment and some natural components	High, but risk of antibiotic resistance with prolonged use	
Biocompatibility	Highly biocompatible, natural, and sustainable; low risk of adverse reactions	Generally biocompatible, but synthetic nature may cause sensitivity in some individuals	Moderate potential cytotoxicity to human cells with prolonged use	Generally biocompatible, derived from natural sources; low risk of adverse reactions	Moderate risk of allergic reactions and skin irritation with prolonged use	
Safety Profile	Safe for most patients; low cytotoxicity, especially in natural formulations	Safe for general use, though some risk of skin sensitivity exists	Potentially cytotoxic; may delay healing in non-infected wounds	Safe for most patients; low risk of adverse effects	Risk of allergic reactions, skin irritation, and antibiotic resistance	
Sustainability	Eco-friendly and sustainable; derived from natural, renewable resources	Less sustainable; often derived from synthetic polymers	Less sustainable; concerns over metal accumulation in the environment	Generally sustainable; derived from natural seaweed sources	Less sustainable; production and overuse contribute to environmental and health concerns	
Cost	Potentially cost-effective due to natural sourcing and less processing required	Moderate; costs vary depending on brand and formulation	Higher cost due to silver content and manufacturing processes	Moderate; costs vary depending on brand and specific formulation	Variable; generally low cost, but long-term use can add up	
